# Telomeric *ORFs* (*TLO*s) in *Candida* spp. Encode Mediator Subunits That Regulate Distinct Virulence Traits

**DOI:** 10.1371/journal.pgen.1004658

**Published:** 2014-10-30

**Authors:** John Haran, Hannah Boyle, Karsten Hokamp, Tim Yeomans, Zhongle Liu, Michael Church, Alastair B. Fleming, Matthew Z. Anderson, Judith Berman, Lawrence C. Myers, Derek J. Sullivan, Gary P. Moran

**Affiliations:** 1 Division of Oral Biosciences, Dublin Dental University Hospital, University of Dublin, Trinity College Dublin, Dublin, Ireland; 2 School of Genetics and Microbiology, University of Dublin, Trinity College Dublin, Dublin, Ireland; 3 Department of Biochemistry, Geisel School of Medicine at Dartmouth, Hanover, New Hampshire, United States of America; 4 Department of Genetics, Cell Biology and Development, University of Minnesota, Minneapolis, Minnesota, United States of America; 5 Department of Microbiology and Biotechnology, George S. Wise Faculty of Life Sciences, Tel Aviv University, Ramat Aviv, Israel; University College Dublin, Ireland

## Abstract

The *TLO* genes are a family of telomere-associated ORFs in the fungal pathogens *Candida albicans* and *C. dubliniensis* that encode a subunit of the Mediator complex with homology to Med2. The more virulent pathogen *C. albicans* has 15 copies of the gene whereas the less pathogenic species *C. dubliniensis* has only two (*CdTLO1* and *CdTLO2*). In this study we used *C. dubliniensis* as a model to investigate the role of *TLO* genes in regulating virulence and also to determine whether *TLO* paralogs have evolved to regulate distinct functions. A *C. dubliniensis tlo1*Δ/*tlo2*Δ mutant is unable to form true hyphae, has longer doubling times in galactose broth, is more susceptible to oxidative stress and forms increased levels of biofilm. Transcript profiling of the *tlo1*Δ/*tlo2*Δ mutant revealed increased expression of starvation responses in rich medium and retarded expression of hypha-induced transcripts in serum. ChIP studies indicated that Tlo1 binds to many ORFs including genes that exhibit high and low expression levels under the conditions analyzed. The altered expression of these genes in the *tlo1*Δ/*tlo2*Δ null mutant indicates roles for Tlo proteins in transcriptional activation and repression. Complementation of the *tlo1*Δ/*tlo2*Δ mutant with *TLO1*, but not *TLO2*, restored wild-type filamentous growth, whereas only *TLO2* fully suppressed biofilm growth. Complementation with *TLO1* also had a greater effect on doubling times in galactose broth. The different abilities of *TLO1* and *TLO2* to restore wild-type functions was supported by transcript profiling studies that showed that only *TLO1* restored expression of hypha-specific genes (*UME6, SOD5*) and galactose utilisation genes (*GAL1* and *GAL10*), whereas *TLO2* restored repression of starvation-induced gene transcription. Thus, Tlo/Med2 paralogs encoding Mediator subunits regulate different virulence properties in *Candida* spp. and their expansion may account for the increased adaptability of *C. albicans* relative to other *Candida* species.

## Author Summary

*Candida albicans* and *C. dubliniensis* are fungal pathogens of humans. Both species possess *TLO* genes encoding proteins with homology to the Med2 subunit of Mediator. The more virulent pathogen *C. albicans* has 15 copies of the *TLO* gene whereas the less pathogenic species *C. dubliniensis* has only two (*TLO1* and *TLO2*). In this study we show that a *C. dubliniensis* mutant missing both *TLO1* and *TLO2* is defective in virulence functions, including hyphal growth and stress responses but forms increased levels of biofilm. Analysis of gene expression in the *tlo1*Δ/*tlo2*Δ mutant revealed extensive differences relative to wild-type cells, including aberrant expression of starvation responses in nutrient-rich medium and retarded expression of hypha-induced transcripts in serum. Tlo1 protein was found to interact with genes and this was associated with both gene activation and repression. *TLO1* was found to be better at restoring hyphal growth compared to *TLO2* and but was less effective than *TLO2* in supressing biofilm formation in the *tlo1*Δ/*tlo2*Δ strain. Thus, Tlo proteins regulate many virulence properties in *Candida *spp. and the expansion of the *TLO* family in *C. albicans* may account for the increased adaptability of this species relative to other *Candida* species.

## Introduction

*Candida albicans* is a commensal yeast commonly recovered from mucosal surfaces in humans. *C. albicans* is also a versatile opportunistic pathogen, responsible for a variety of superficial infections as well as more severe, life threatening infections in severely immunocompromised patients. Phenotypic versatility is an important characteristic of *C. albicans* and this flexibility allows *C. albicans* to adapt to this wide range of niches in the human host [1]–[3]. Over the past decade, transcriptional profiling of *C. albicans* using microarray and RNA-seq technologies has revealed that rapid adaptation to local environmental conditions involves elaborate, programmed shifts in transcription pattern. The biochemistry of transcriptional regulation has been studied intensively in the model yeast *S. cerevisiae*. Most genes transcribed by RNA polymerase II (PolII) require a carefully orchestrated series of events to initiate transcription. The formation of the pre-initiation complex and recruitment of PolII requires the function of Mediator, a large multi-subunit protein complex [4], [5]. Mediator is thought to be required to bridge DNA-bound transcription factors with the rest of the transcriptional machinery [6], [7]. The complex is generally divided into three modules: a head, middle and tail. The tail region includes Med2, Med3 and Med15/Gal11 and is thought to be the part of Mediator that interacts directly with transcription factors such as Gal4 and Gcn4 [8].

Most components of Mediator are highly conserved between *S. cerevisiae* and *C. albicans* [9]. However, completion of the *C. albicans* genome sequence [10], [11] revealed a family of up to 15 telomeric (*TLO*) genes that were subsequently found to encode a domain with homology to the Mediator tail subunit Med2 of *S. cerevisiae* [9], [12], [13]. This *TLO* gene expansion is unique to *C. albicans*, as most of the less pathogenic non-*albicans Candida* species such as *C. tropicalis* and *C. parapsilosis* have only one *TLO* gene. *C. dubliniensis*, the closest relative to *C. albicans* has two *TLO* genes, namely *TLO1* and *TLO2*. *TLO1* (*Cd36_72860*) is located internally on chromosome 7 and *TLO2* (*Cd36_35580*) is at a telomere-adjacent locus on the right arm of chromosome R [14]. *C. dubliniensis TLO2* appears to be the ancestral locus, based on synteny with, and homology to, the single *C. tropicalis* orthologue CTRG_05798.3 [14]. All candidal *TLO* genes share the Med2-like domain of the *C. albicans TLOs* and appear to be the *MED2* orthologs in these species. Indeed, biochemical studies have shown that like the *S. cerevisiae* Med2 protein, Tlo proteins co-purify with the *C. albicans* orthologs of mediator tail module components Med3 and Med15 [9].

The 15 *TLO* genes in the *C. albicans* type strain SC5314 can be classified into four distinct clades based on gene structure, including the highly expressed *TLO*α clade (6 members), a single *TLO*β gene, the poorly expressed *TLO*γ clade (7 members) and a single *TLO*ψ gene which is a pseudogene [12]. Biochemical studies have shown that the levels of Tloα and Tloβ proteins is in vast excess to the amount necessary to be a stoichiometric subunit of Mediator (9) and stands in stark contrast to the situation in *S. cerevisiae*, where the Med2 subunit is expressed at roughly equivalent amounts to other subunits of the complex. Due to the large numbers of *TLO* genes in *C. albicans*, functional analysis of Mediator has largely been restricted to other subunits, including Med3 (tail domain), Med31 (middle domain), Med20 (head domain) and Med13/Srb9 (kinase domain). Med31 and Med20 are required for the transition of *C. albicans* yeast cells to filamentous hyphae and for biofilm formation, two important pathogenic traits of the organism [9], [15]. Med31 is required for expression of the genes *ALS1* and *ALS3* that encode cell-surface proteins involved in biofilm production, consistent with the poor biofilms produced by *med31*Δ mutants. In addition, Med31 is required for the expression of genes regulated by the transcription factor *Ace2*, which regulates many genes involved in cell wall remodelling during cell separation. Consistent with this, the *med31*Δ mutant exhibits defective cytokinesis. Deletion of *MED3* resulted in a stronger filamentous growth defect, resulting in short pseudohyphae in serum or liquid media supplemented with GlcNac [9]. Deletion of Mediator subunits also has complex effects on the transcriptional circuit governing the white-opaque switch, a phenotypic transition involved in mating [16].

*C. albicans* is the most prevalent pathogenic fungal species. The closely related species *C. dubliniensis* is responsible for far fewer infections and is a minor component of the human oral flora [14], [17]. The yeast-to-hypha transition is an important virulence trait of *C. albicans* and *C. dubliniensis* forms fewer hyphae than *C. albicans* in response to most *in vitro* and *in vivo* hypha-inducing stimuli [17]. *C. dubliniensis* requires much stronger environmental cues, including nutrient depletion, in order to activate a transcriptional response that will induce the yeast-to-hypha transition [18]–[20]. Less effective transcriptional responses may also account for the increased susceptibility of *C. dubliniensis* to environmental stress relative to *C. albicans* [21]. It has been suggested that the expansion of the *TLO* family in *C. albicans* may account for the greater transcriptional flexibility and adaptability of this species relative to *C. dubliniensis* and the other non-*albicans Candida* species [22]. However, direct analysis of *TLO* null mutants has not been attempted in *C. albicans*, due to the large number of *TLO* genes and the likelihood of redundancy in the *TLO* family.

In this study, we exploited the closely related species *C. dubliniensis* as a model to study *TLO* gene function and to construct the first *tlo* null strains. We found that *C. dubliniensis* mutants lacking both *TLO1* and *TLO2* (*tlo1Δ/tlo2Δ*) exhibit widespread changes in the transcription of virulence-associated genes. ChIP analysis detected Tlo1 within the coding regions of ORFS, as well as subtelomeric regions and the Major Repeat Sequence (MRS). Interestingly, in strains lacking only one of the two *TLO* genes, expression of distinct subsets of genes was altered. We propose that expansion of the *TLO* family, even to only two members, has facilitated the evolution of functional diversity and may be of particular importance in the evolution of an expanded set of *TLO* paralogs together with the increased virulence in *Candida albicans*.

## Results

### *Candida dubliniensis* possesses two expressed *TLO* paralogs

**Figure 1:**
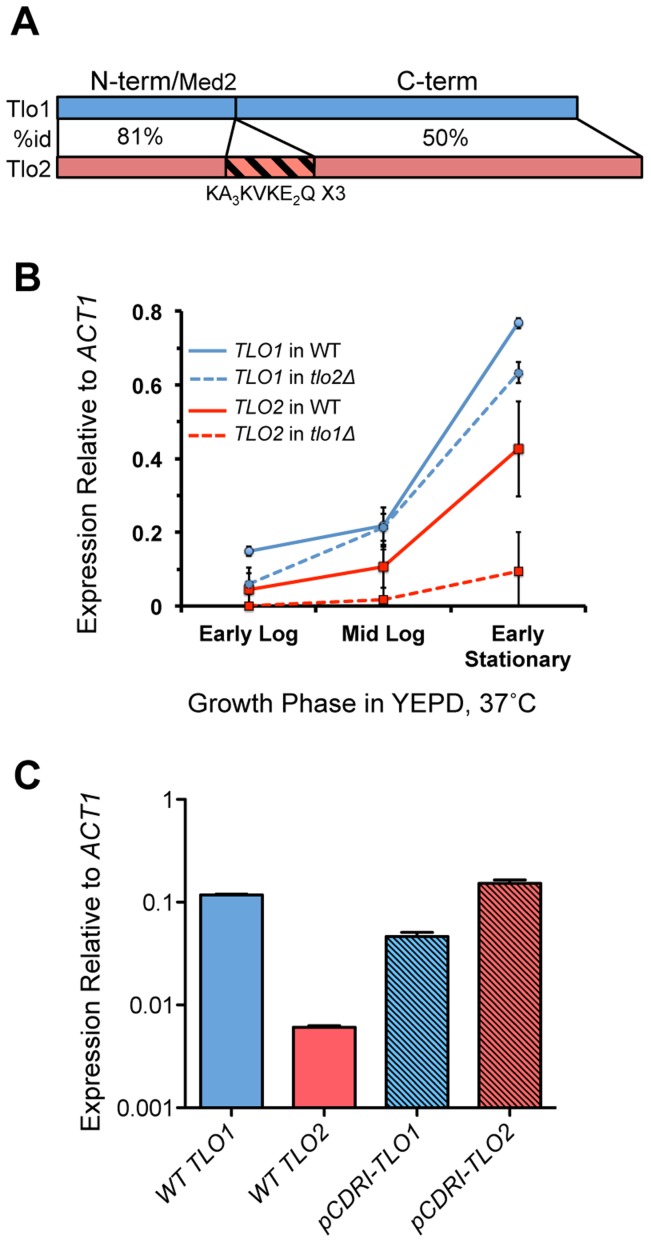
Structure and expression of *C. dubliniensis TLO* genes. (A) *TLO1* and *TLO2* encode proteins of 320 and 355 amino acids respectively that exhibit 81% identity in the N-terminal Med2-like domain, and 50% identity in the C-terminal portion of the protein. In addition, Tlo2 has an internal triplet repeat, indicated. (B) Expression of *TLO1* and *TLO2* was measured by QRT-PCR using gene-specific primers during growth in YEPD broth at 37°C in early exponential (1.5 h-post inoculation), mid-exponential (8 h) and early stationary phase (18 h) in strain Wü284 (WT). The effect of deletion of either *TLO1* or *TLO2* on the expression of the other paralog was also analysed. (C) Expression of reintegrated copies of *TLO1* and *TLO2* in plasmid pCDRI in the *tlo1*Δ/*tlo2*Δ strain.

The two *Candida dubliniensis TLO* genes, *TLO1* and *TLO2*, encode proteins of 320 and 355 amino acids, respectively, and share 58% identity. Homology to each other and to the *C. albicans* Tlo proteins is concentrated in the N-terminal 120 residues of the proteins. Tlo1 and Tlo2 share 81% identity in this N-terminal region, which also exhibits homology to *S. cerevisiae* Med2 (Figure 1A). The amino acid sequence of Tlo2 is 25 residues longer and contains a central triplet repeat of the motif KAAAKVKEEQ. *C. dubliniensis* is a diploid organism and gene deletion studies (see below) and Southern blot analysis confirmed that *C. dubliniensis* strain Wü284 has two alleles of *TLO1* (Figure S1). However, Southern blot experiments could detect only one allele of *TLO2* at the telomere of chromosome R (ChR) in strain Wü284, indicating that one copy of ChR in Wü284 was truncated resulting in loss of one allele of *TLO2* (Figure S1). PCR analysis of the *tlo2Δ* strain confirmed that bases 1 to 918 of the second *TLO2* allele are deleted due to this truncation, with only a small 150 bp remnant of the ORF remaining. Analysis of gene expression by QRT-PCR revealed that *TLO1* mRNA is expressed over 2-fold higher than *TLO2* mRNA in YEPD at 37°C (Figure 1B).

### *C. dubliniensis* Tlo proteins and Med3 are components of the Mediator tail module

**Figure 2:**
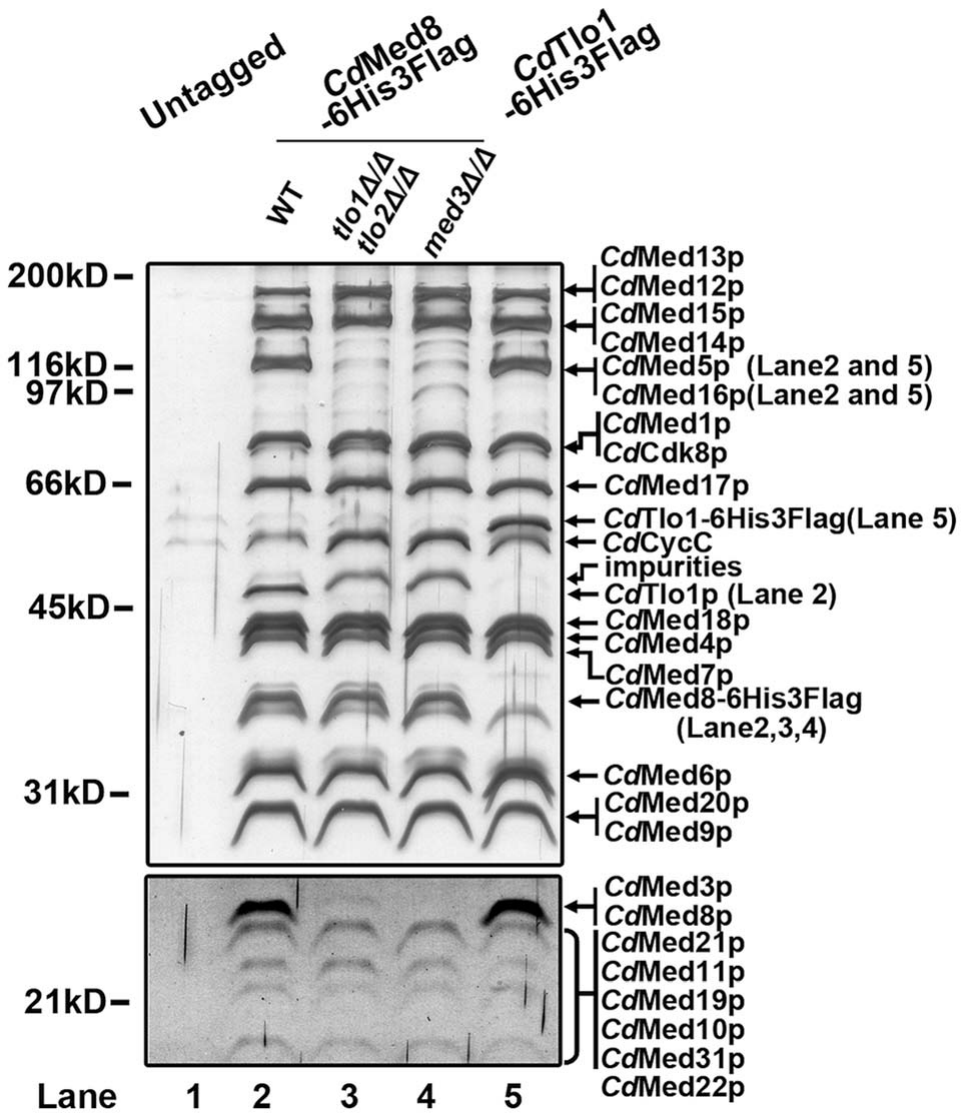
Biochemical analysis of Mediator in *C. dubliniensis* wild-type and *tlo1*Δ/*tlo2*Δ. Lysates from untagged WT *C. dubliniensis* (Lane 1), a Med8-6His-Flag-tagged WT strain (Lane 2), a Med8-6His-Flag-tagged *tlo1*Δ/*tlo2*Δ strain (Lane 3), a Med8-6His-Flag-tagged *med3*Δ strain (Lane 4) and a Tlo1-6His-Flag-tagged WT strain (Lane 5) were subjected, in parallel, to multiple chromatographic separations. The elutions from the IMAC (Talon) step were analyzed by 8% SDS-PAGE. Proteins were revealed by staining with silver. Bands that are present in WT but clearly absent in the *tlo1*Δ/*tlo2*Δ and *med3*Δ Mediator are indicated in parenthesis on the right. The absence of these proteins in the *tlo1*Δ/*tlo2*Δ Mediator was confirmed by mass spectroscopy.

We first asked if *C. dubliniensis* Tlo is a component of Mediator. To address this, we purified Mediator from whole cell extracts of *C. dubliniensis* wild-type and *tlo1Δ/tlo2Δ* mutant cells using a dual affinity tag on the *C. dubliniensis* ortholog of the conserved Med8 subunit of Mediator. The resulting Mediator complex was analyzed for purity by SDS-PAGE and for composition by mass spectroscopy with untagged WT *C. dubliniensis* serving as a control. Biochemical analysis showed that the *C. dubliniensis* Mediator, like *C. albicans*, is composed of a complete set of orthologs of the *S. cerevisiae* complex (Figure 2). Mediator purified from the *tlo1*Δ/*tlo2*Δ mutant lacked tail subunits Med3, Med15, Med16 and Med5 subunits (Figure 2, Lane 3). Importantly, Mediator purified from the *C. dubliniensis tlo1*Δ/*tlo2*Δ strain or from a *med3*Δ null strain had equivalent composition, lacking tail components Tlo1, Med3, Med5, Med15 and Med16 (Figure 2, Lanes 3 and 4).

To determine the relative abundance of Tlo proteins compared to other Mediator subunits, we constructed strains carrying Tlo1-HA, Med3-HA and Med8-HA tagged derivatives. Immunoblotting of whole cell extracts revealed that *C. dubliniensis* Tlo1 Mediator subunit is expressed in amounts comparable to the Med3 Tail Module and Med8 Head Module subunits (Figure S2). Consistent with this finding, purification of an affinity tagged Tlo1 protein from *C. dubliniensis* yielded only the Mediator associated form (Figure 2, Lane 5) and no free Tlo1 protein. Thus, in *C. dubliniensis* Tlo1 appears to be acting solely as a component of Mediator.

### *TLO1* activates hyphal growth to a greater extent than *TLO2*

**Figure 3:**
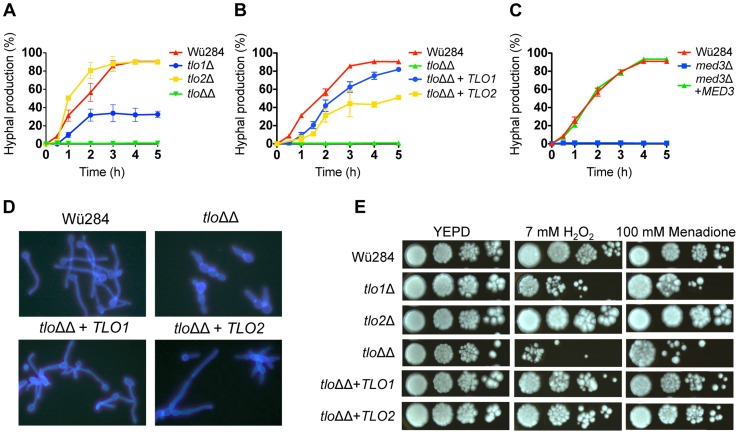
Phenotypic analysis of *C. dubliniensis tloΔ* and *med3*Δ mutants. The rate of formation of true hyphae in water supplemented with 10% serum was measured in the *tlo1*Δ, *tlo2*Δ and *tlo1*Δ/*tlo2*Δ (*tlo*ΔΔ) mutants relative to Wü284 (A), in the *TLO1* and *TLO2* reintegrants (*tlo*ΔΔ+*TLO1/2*)(B) and in the *med3*Δ mutant (C). Measurements were taken over a period of 5 h by counting the number of cells producing unconstricted germ-tubes and expressing this value as a percentage of the whole population. (D) Cellular morphology of Wü284, the *tlo1*Δ/*tlo2*Δ (*tlo*ΔΔ) mutant and *TLO1* and *TLO2* reintegrated strains in 10% serum following staining with calcofluor white. (E) Oxidative stress susceptibility in *tlo*Δ mutants. Susceptibility to oxidative stress was assessed by spotting 20 µl aliquots of 10-fold serially diluted suspensions (10^6^ to 10^3^ cells/ml) of the indicated strains on YEPD agar plates containing 7 mM H_2_O_2_ or 100 mM menadione. Plates were incubated for 48 h at 37°C.

Deletion of *TLO2* did not reduce filamentous growth significantly (Figure 3A), while deletion of *TLO1* resulted in reduced hyphal production (Figure 3A), as reported previously [14]. However, the double *tlo1*Δ/*tlo2*Δ mutant (*tlo*ΔΔ) had a more severe filamentation defect than the *TLO1* deletion alone, suggesting that *TLO2* partly compensates for the deletion of *TLO1* in the single mutant (Figure 3A, 3D). The cellular morphology of the *tlo1*Δ/*tlo2*Δ mutant in 10% serum was characteristically pseudohyphal (Figure 3D).

In order to investigate if *TLO1* and *TLO2* are functionally different, we reintroduced *TLO1* and *TLO2* individually to the *tlo1*Δ/*tlo2*Δ mutant using a previously described integrating vector pCDRI. This strategy was employed as it allowed us to express both *TLO1* and *TLO2* to similar levels (Figure 1C) thus allowing a direct comparison of their ability to complement the phenotypes of the *tlo1*Δ/*tlo2*Δ mutant. Introduction of *TLO1* could restore true hypha production to almost wild-type levels, whereas *TLO2* restored hypha formation to approximately 50% of wild-type levels (Figure 3B, 3D). These data show that *TLO1* and *TLO2* differ in their ability to activate filamentous growth.

### Deletion of *TLO1* and *TLO2* produces multiple phenotypes

The colony morphology of the *tlo1*Δ, *tlo2*Δ and *tlo1*Δ/*tlo2*Δ mutant strains following growth on nutrient-rich solid medium (YEPD agar) at 37°C was indistinguishable from that of wild-type. At the cellular level, the *tlo1*Δ/*tlo2*Δ mutant grew as short chains of 3–4 cells, which is indicative of a defect in cytokinesis (Figure S3A). A similar phenotype was described by Uwamahoro et al. [15] in a *C. albicans med31*Δ mutant. Wild-type cytokinesis was restored in the *tlo1*Δ/*tlo2*Δ mutant following reintroduction of *TLO1* but not *TLO2* (Figure S3A). Growth defects of *tlo* mutants became apparent when grown on non-optimal media. On Spider agar, the wild-type strain formed smooth white colonies, typical of *C. dubliniensis* on this medium. However, the *tlo1*Δ/*tlo2*Δ mutant formed heavily wrinkled colonies on this medium (Figure S3B). Wild-type smooth colonies could be restored by complementation of the *tlo1*Δ/*tlo2*Δ mutant with either *TLO1* or *TLO2* (Figure S3B). Following 5 days incubation on Pal's medium, the double mutant produced extensive levels of pseudohyphae, but unlike wild-type which produced copious amounts of chlamydospores on this medium, no terminal differentiation of these pseudohyphae to chlamydospores was observed (Figure S4A). The *TLO1* mutant and the *tlo1*Δ/*tlo2*Δ double mutant also exhibited longer doubling times in several different liquid media, including YEPD (Table 1). When galactose was substituted for glucose in YEP medium (YEP-GAL), the growth rate of the *tlo1Δ* and the *tlo1*Δ/*tlo2*Δ mutants was reduced with doubling times 1.4 to 1.6 times longer than wild-type doubling times, respectively (Table 1). Growth in synthetic liquid Lee's medium was also retarded, with a doubling time 1.6 fold higher than that of wild-type. The addition of 1% peptone restored growth to WT levels in the *tlo1*Δ/*tlo2*Δ mutant (Figure S4B) while the addition of carbon sources or individual amino acids to solid Lee's medium did not.

**Table 1:** Doubling times (min) of *tlo*Δ mutants and complemented strains *in vitro*.*

Growth Media	Wü284	*tlo1*Δ	*tlo2*Δ	*tlo*ΔΔ	*tlo*ΔΔ+*TLO1*	*tlo*ΔΔ+*TLO2*
**YEPD**	94.92	104.6	91.72	102.9	*nd*	*nd*
	*0.9865*	*0.9502*	*0.9783*	*0.9044*	*nd*	*nd*
**YEP-Galactose**	95.17	129.2	106.8	149.3	84.29	103.1
	*0.9828*	*0.9933*	*0.9995*	*0.9821*	*0.9916*	*0.9974*
**YEP-Succinate**	89.31	110.8	91.88	132	94.46	112
	*0.9228*	*0.9835*	*0.9826*	*0.9037*	*0.9707*	*0.8614*
**Lee's defined medium**	100.6	106.6	97.27	157.6	127.3	130.1
	*0.978*	*0.9598*	*0.9836*	*0.7243*	*0.8682*	*0.9675*
**Spider medium**	152.4	168.2	145.4	178.7	161.1	175.9
	*0.9535*	*0.9842*	*0.9528*	*0.966*	*0.9464*	*0.9456*

* The coefficient of determination (r^2^) value is shown italicised below each doubling time value as an indicator of confidence in the data when analysed using regression.

Reintroduction of either *TLO1* or *TLO2* in the *tlo1Δ/tlo2Δ* double mutant background restored doubling times to wild-type levels in YEP-GAL. However, reintroduction of *TLO1* had a significantly greater effect on doubling time compared to complementation with *TLO2*. In contrast, either *TLO1* or *TLO2* restored the growth defect on Lee's medium to the same degree (Table 1).

We next investigated if *TLO* genes affect a range of stress responses. Supplementation of YEPD agar with H_2_O_2_ (7 mM) or menadione (100 mM), which generate reactive oxygen species, inhibited growth of the *tlo1Δ/tlo2Δ* mutant, partly inhibited growth of the *tlo1Δ* mutant, while growth of the *tlo2Δ* single mutant was largely unaffected by these oxidizing agents (Figure 3E). Reintroduction of either *TLO1* or *TLO2* to the *tlo1Δ/tlo2Δ* strain restored wild type growth in the presence of both H_2_O_2_ and menadione, indicating that both *TLO* genes have a role in the growth of cells under oxidative stress.

### *med3Δ* phenocopies a *tlo1Δ/tlo2Δ* mutant

Tlo proteins are components of the Mediator complex, which is thought to facilitate interactions between Mediator and DNA-bound transcriptional activators [8]. As Tlo protein and Med3 are required for the stability of the Mediator tail in *C. dubliniensis* (Figure 2), we hypothesized that deletion of *MED3* and *TLO* genes should result in similar phenotypic effects. To test this, we compared the phenotypes of *C. dubliniensis tlo1Δ/tlo2Δ* and *med3Δ* mutants. The *C. dubliniensis med3*Δ mutant, similar to the *C. albicans med3*Δ mutant [9] and the *C. dubliniensis tlo1Δ/tlo2Δ* mutant was unable to form true hyphae in 10% serum (Figure 3C) and exhibited extended doubling times in YEP-Gal (Figure S5A) and increased susceptibility to H_2_O_2_ (Figure S5B). All of these properties could be restored to wild-type levels in the *med3*Δ mutant with the reintroduction of a wild-type copy of *MED3* on pCDRI (Figure 3C, S4A, S4B).

### Tlo proteins regulate the transcription of a diverse set of genes

**Figure 4:**
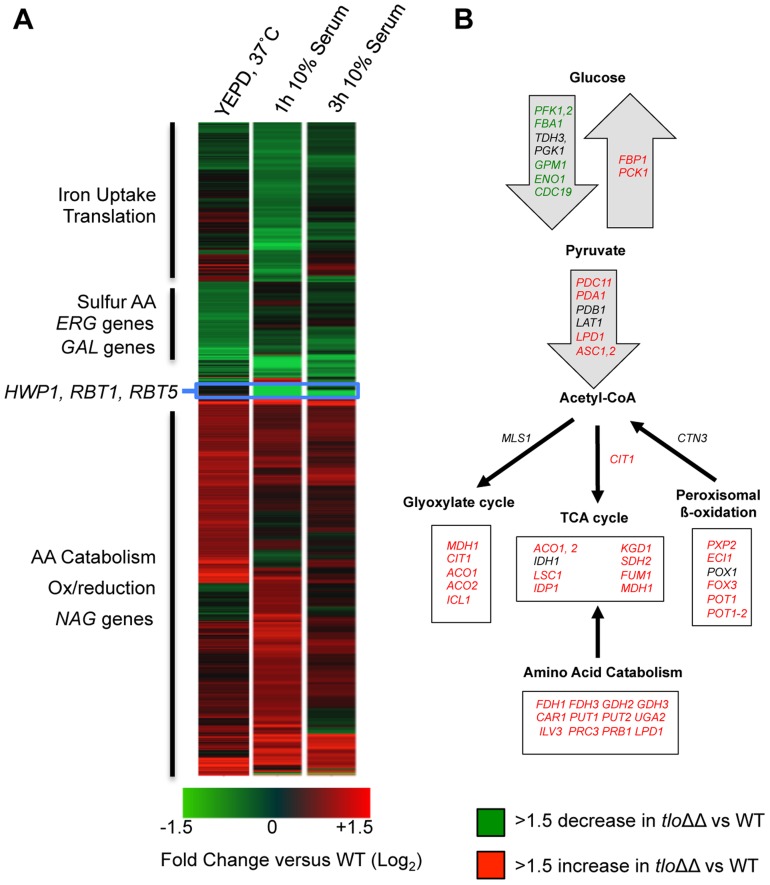
Microarray gene expression profiling of the *tlo1*Δ/*tlo2*Δ (*tlo*ΔΔ) mutant in YEPD and 10% serum. (A) A heat map generated in GeneSpring GX12 showing all 1.5 fold regulated genes in the *tlo1*Δ/*tlo2*Δ (*tlo*ΔΔ) mutant relative to wild type strain Wü284 during exponential growth in YEPD broth and following inoculation in 10% (v/v) serum (1 h and 3 h). The fold change (Log_2_) relative to wild-type is color coded as indicated in the lower panel. GO terms associated with up and down regulated clusters are indicated on the left. (B) A cartoon metabolic map of the *tlo1*Δ/*tlo2*Δ (*tlo*ΔΔ) mutant showing the changes in expression of genes involved in energy metabolism during growth in YEPD. Genes in green exhibit a 1.5-fold or greater reduction in expression relative to wild-type whereas those in red exhibited a 1.5-fold increase in expression. Genes in black were not significantly changed.

Since Mediator is important for transcription regulation, we analysed RNA expression patterns in the *tlo1*Δ/*tlo2*Δ mutant relative to wild-type cells grown in nutrient-rich growth conditions (YEPD at 37°C) and grown in hyphal inducting conditions (water plus 10% serum, optimal for *C. dubliniensis* hypha formation). During exponential growth in YEPD, a total of 746 genes exhibited a 1.5-fold or greater increase in expression and 635 genes exhibited a 1.5-fold or greater reduction in expression (Q≤0.05; Figure 4A). This scale of differential gene expression observed in our *tlo1Δ/tlo2Δ* is similar to that seen in *S. cerevisiae* Mediator tail mutants [23], [24]. In the nutrient-rich YEPD broth, the *tlo1Δ/tlo2Δ* mutant exhibited a transcriptional profile that resembled a response to nutrient starvation (Figure 4B). The induced set of genes was enriched for processes associated with catabolism of alternative carbon and nitrogen sources such as *N*-acetyl-glucosamine (*NAG1, NAG3, NAG4, NAG6*), amino acids (e.g. *GDH2*, *CAR1*, *PUT2*, *PUT1*, *LPD1*, *FDH1* and *FDH3*) and fatty acids. The *tlo1*Δ/*tlo2*Δ mutant cells also up-regulated key genes of gluconeogenesis (*PCK1* and *FBP1*) and the glyoxylate cycle (*ICL1* and *MDH1*) (Figure 4B). In concert with this, the *tlo1*Δ/*tlo2*Δ mutant also caused down-regulation of genes encoding glycolytic enzymes (*PFK1*, *PFK2*, *FBA1*, *GPM1* and *ENO1*) and the glycolytic regulator *TYE7*. The *tlo1*Δ/*tlo2*Δ mutant also exhibited a greater than 2-fold decrease in expression of genes encoding proteins important for sulphur amino acid biosynthesis (e.g. *SAM2, MET1, MET6, MET10, MET14, MET16*) and ergosterol biosynthesis (*ERG1, ERG9, ERG25*). In addition, some hypha-specific genes were induced in the *tlo1*Δ/*tlo2*Δ mutant grown in YEPD. This included *IHD1*, *RBT5* and *SAP7* (induced in *C. dubliniensis* hyphae) as well as several regulators of biofilm and hyphal growth (*BCR1*, *NRG1*, *SFL1*, *TEC1* and *EED1*).

Gene Set Enrichment Analysis (GSEA) was used to compare the *tlo1*Δ/*tlo2*Δ regulated genes with published microarray datasets. GSEA indicated that the set of genes differentially expressed in the *tlo1*Δ/*tlo2*Δ mutant was enriched for genes induced during infection of RHE [25] and genes both induced and repressed during infection of bone-marrow derived macrophages [26] (Figure S6).

We also examined the transcript profile of the *tlo1Δ/tlo2Δ* mutant in 10% serum relative to wild-type (Figure 4A). After 1 h, 868 genes were significantly up-regulated 1.5-fold or greater and 816 ORFs downregulated (Q≤0.05) relative to wild-type expression at the same time point. In this medium the *tlo1Δ/tlo2Δ* mutant exhibited reduced expression of several key regulators of filamentous growth including *RAS1*, *RIM101*, *EFG1* and *UME6*. In addition, the hyphal growth regulating cyclins *CLN3* and *HGC1* were also down regulated in the *tlo1Δ/tlo2Δ* mutant. The *tlo1Δ/tlo2Δ* mutant also exhibited reduced expression of many cell wall proteins whose induction is chracteristic of the yeast-to-hypha transition in *C. dubliniensis*, including *HWP1*, *RBT1*, *RBT5* and *SOD5* [20]. Paradoxically the *tlo1*Δ/*tlo2*Δ mutant exhibited a more rapid induction of the hypha-specific gene *ECE1* following 1 h incubation in serum, and expression remained elevated relative to wild-type at 3 h. In addition to hyphal genes, the *tlo1Δ/tlo2Δ* mutant exhibited reduced expression of galactose metabolic genes *GAL1*, *GAL7* and *GAL10*.

After 3 hours in 10% serum, a similar number of genes were differentially expressed 1.5-fold or greater (n = 1726). However, the level of differential expression at many genes had decreased by 3 h (Figure 4A).

### *TLO1* and *TLO2* restore expression of different subsets of genes

**Figure 5:**
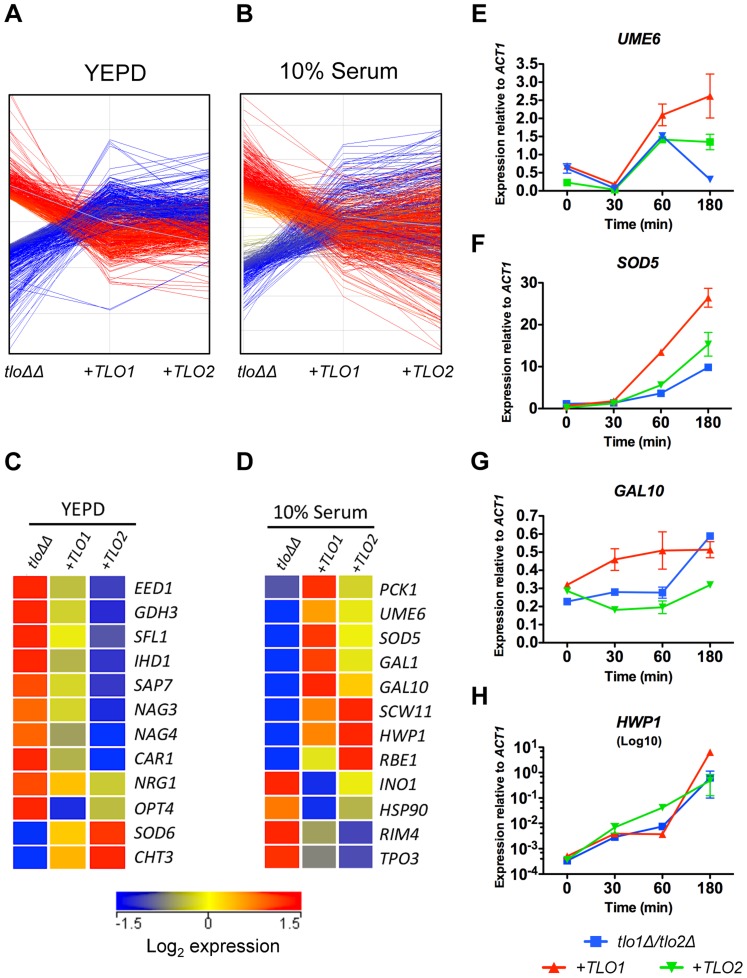
Reintroduction of *TLO1* or *TLO2* in the *tlo1*Δ/*tlo2*Δ (*tlo*ΔΔ) mutant restores wild-type levels of expression of overlapping and distinct sets of genes. (A) A graph plotting the change in expression of genes up regulated (red lines) or down regulated (blue lines) in the *tlo1*Δ/*tlo2*Δ (*tlo*ΔΔ) mutant following complementation with either *TLO1* (*+TLO1*) or *TLO2* (*+TLO2*) in YEPD or (B) 10% (v/v) serum. (C) Heat map of selected genes that were differentially expressed in the *tlo1*Δ/*tlo2*Δ (*tlo*ΔΔ) mutant during growth in YEPD broth (C) or 10% (v/v) serum (D) that exhibited a *TLO1* or *TLO2* specific restoration to wild-type in expression. Colors in the first column (*tlo1*Δ/*tlo2*Δ) represent the fold change in expression of the indicated genes in *tlo*ΔΔ vs wild-type; colors in columns 2 and 3 (+*TLO1* and +*TLO2*) represent the fold change in expression of the indicated genes following reintegration of *TLO1* or *TLO2* versus *tlo*ΔΔ. (E to H) QRT-PCR of the indicated genes was carried out following 30, 60 and 180 min incubation in 10% (v/v) serum at 37°C. Gene expression was determined using Sybr green technology and gene-specific levels are expressed relative to *ACT1*. Primer sequences are listed in Table S5.

In order to investigate whether *TLO1* and *TLO2* regulate similar or different sets of genes, we analysed the transcript profiles of the *TLO1* and *TLO2* complemented mutants rather than the *tlo1*Δ and *tlo2*Δ single mutants, as the complemented strains exhibited equivalent expression of *TLO1* or *TLO2* (Figure 1B, 1C). Both *TLO1* and *TLO2* largely restored the transcript profile of the *tlo1*Δ/*tlo2*Δ mutant back to wild-type levels in both YEPD and 10% serum (Figure 5A, 5B). However, some *TLO*-specific transcript patterns were also detectable. Sixty-one genes were regulated in a *TLO*-specific manner in YEPD (Table S1). In comparison to the *TLO1*-complemented strain, the *TLO2*-complemented strain showed a greater reduction in expression of many *tlo1*Δ/*tlo2*Δ induced genes, including many hyphal genes (*IHD1*, *SAP7* and *EED1*), negative regulators of hyphal growth (*NRG1* and *SFL1*) and some starvation-induced genes such as *CAR1*, *GDH3*, *NAG3* and *NAG4* (Figure 5C). The *TLO2* complemented strain was also better at restoring wild-type levels of *SOD6* expression relative to the *TLO1* complemented strain (Figure 5C).

In 10% serum, *TLO1* was more effective at restoring expression of several genes required for induction of, and induced during, filamentous growth including *UME6* and *SOD5* (Table S2, Figure 5D). *TLO1* was also a more effective inducer of *GAL1* and *GAL10* expression, which may explain why restoration of growth in galactose medium was more effective in the *TLO1* reintegrant relative to the *TLO2* complemented strain (Figure 5D). In contrast, *TLO2* was more effective at inducing *HWP1* expression (Figure 5D). More detailed analysis of hyphal-induced gene expression by QRT-PCR corroborated these microarray findings and supported the existence of a temporal defect in the induction of hypha specific genes in the *tlo1*Δ/*tlo2*Δ mutant. QRT-PCR showed that *UME6* and *SOD5* exhibited weak levels of induction in the *tlo1*Δ/*tlo2*Δ mutant relative to the *TLO1* reintegrant (Figure 5E, 5F). Although addition of *TLO2* led to a minor increase in *SOD5* expresssion, complementation with *TLO1* restored high-level induction of *SOD5*. Similarly, induction of *GAL10* also occurred more rapidly in the *TLO1*-reintegrated strain relative to both the *tlo1*Δ/*tlo2*Δ mutant and the *TLO2*-reintegrated strain (Figure 5G) Conversely, QRT-PCR confirmed that *TLO2* restored more rapid induction (∼10-fold) of the *HWP1* transcript following 1 h incubation in serum relative to the *TLO1* reintegrant (Figure 5H). However *HWP1* expression in the *tlo1Δ/tlo2Δ* mutant and *TLO1* reintegrant reached similar levels following three hours incubation in serum (Figure 5H).

This transcriptional analysis of the *TLO1* and *TLO2* complemented strains for the first time demonstrate functional diversification of Tlo proteins and show that different Tlos can regulate distinct subsets of genes.

### Deletion of *MED3* disrupts transcription of a similar set of genes

**Figure 6:**
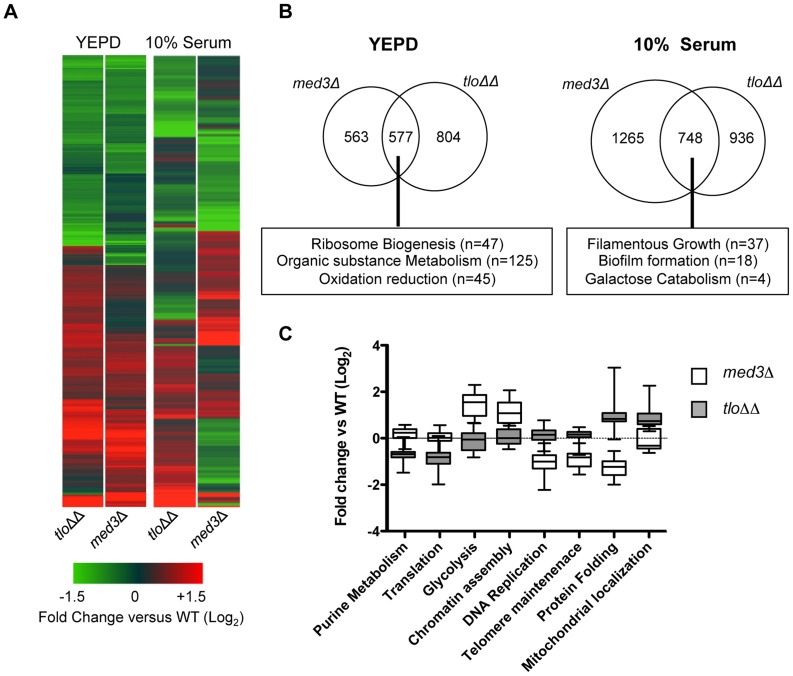
Microarray gene expression profiling of the *med3*Δ mutant in YEPD and 10% (v/v) serum. (A) A heat map generated in GeneSpring GX12 showing all 1.5 fold regulated genes in the *tlo1*Δ/*tlo2*Δ (*tlo*ΔΔ) and *med3*Δ mutants relative to wild type strain Wü284 during exponential growth in YEPD broth and following inoculation in 10% serum (1 h). The fold change (Log_2_) relative to wild-type is color coded as indicated in the lower panel. (B) Venn diagrams illustrating the numbers of genes commonly regulated greater than 1.5-fold in the *tlo1*Δ/*tlo2*Δ (*tlo*ΔΔ) and *med3*Δ mutants following growth in YEPD broth or 10% serum. The box below indicates major functional classes of commonly regulated genes identified by GO term analysis using the GO Term finder at CGD (http://www.candidagenome.org/). (C) Graph plotting the expression levels (Log_2_ fold change versus wild-type) of genes associated with selected GO terms in the *tlo1*Δ/*tlo2*Δ (*tlo*ΔΔ) and *med3*Δ mutants during growth in 10% (v/v) serum.

This study has shown that deletion of *MED3* in *C. dubliniensis* results in similar phenotypes to those observed in the *tlo1*Δ/*tlo2*Δ mutant. In order to determine whether these gene deletions resulted in similar changes in transcript profile, we compared gene expression in the *tlo1*Δ/*tlo2*Δ and the *med3*Δ mutant in YEPD broth and in 10% serum. Hierarchical clustering was used to compare all 1.5-fold regulated genes in strains *tlo1*Δ/*tlo2*Δ and *med3*Δ during exponential growth. This comparison demonstrated the similarity of the transcriptional changes in both mutants relative to wild-type cells (Figure 6A). A large number (n = 47) of these commonly down-regulated genes were associated with the GO term “ribosome biogenesis” (*P* = 3.89×10^−19^; FDR = 0.0). In YEPD, both mutants exhibited aberrantly increased expression of the hypha-specific genes *IDH1*, *RBT5* and *SAP7* as well as the regulators *TEC1* and *EED1*. Activation of starvation responses was also evident in the *med3Δ* strain, which like the *tlo1*Δ/*tlo2*Δ strain exhibited increased expression of genes involved in gluconeogenesis (*PCK1, FBP1*) and the glyoxylate cycle (*ICL1, MDH1*). Decreased expression of glycolytic genes was less pronounced in *med3Δ*, however we could detect decreased expression (>1.5-fold) of *PFK1*, *PFK2*, *CDC19* the glycolytic regulator *TYE7* (Figure S7). Similar to *tlo1*Δ/*tlo2*Δ, *med3*Δ also exhibited increased expression of genes involved in oxidation/reduction processes, including amino acid catabolism (Figure 6B) in addition to reduced expression of genes involved in sulphur amino acid metabolism (*CYS3, MET1, 2, 4, 10*) and ergosterol metabolism (*ERG1, ERG251*).

Following 1 h growth in water plus 10% serum the *med3*Δ and *tlo1*Δ/*tlo2*Δ mutants again exhibited a common set of co-regulated genes, however in serum many *med3*Δ mutant-specific responses could also be identified (Figure 6B, 6C). The common gene set included regulators of filamentous growth, cell wall proteins and galactose metabolic genes (Figure 6B). Importantly, the *med3Δ* mutant specifically exhibited an increase in expression of glycolytic genes (n = 11; Figure 6C, S6) and genes encoding histones and proteins involved in chromatin assembly (n = 18). The *med3*Δ mutant also exhibited reduced transcription of genes associated with the GO terms “DNA replication” (n = 43) and “telomere maintenance” (n = 18) (Figure 6C).

These data indicate that in exponentially growing cells, Tlo proteins and Med3 are involved in regulating very similar processes. However, in response to specific environmental cues, these proteins may be required for regulation of specific sets of genes.

### Deletion of *TLOs* has different effects on virulence traits compared to a *C. albicans med31*Δ mutant

**Figure 7:**
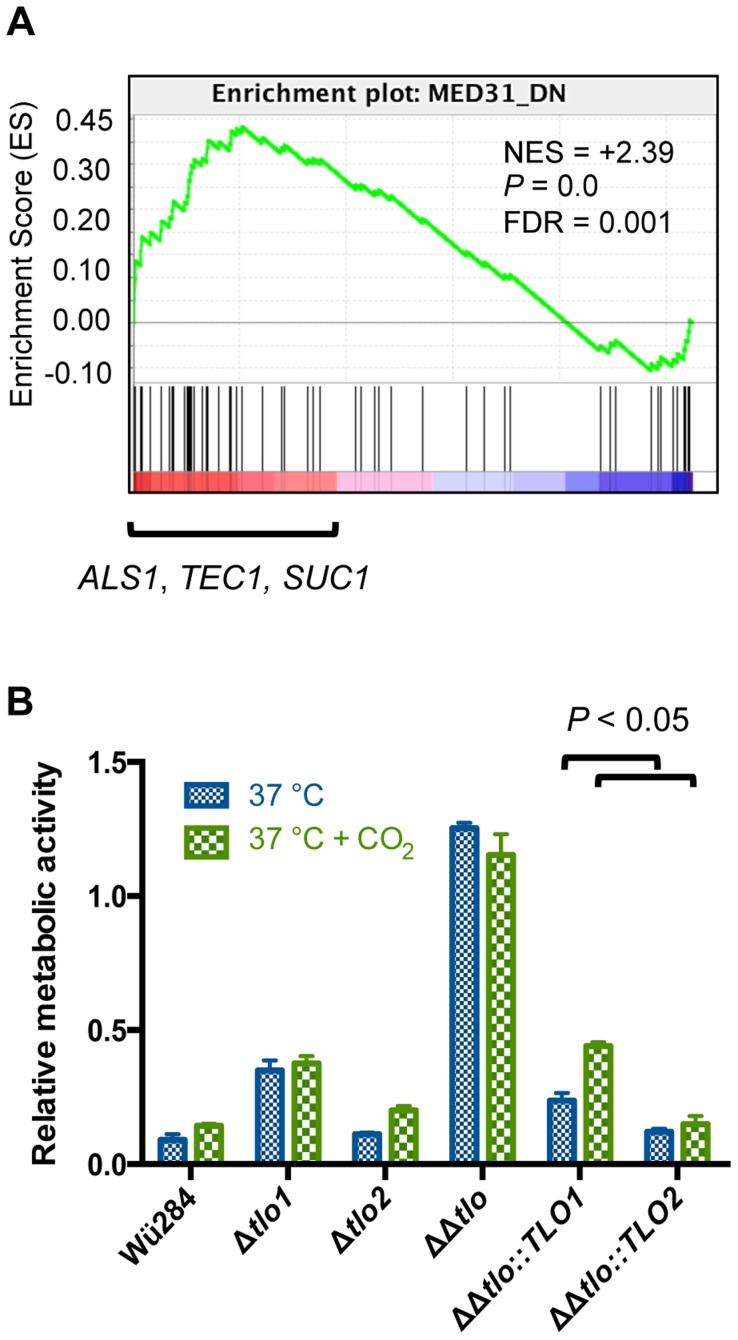
Deletion of *TLOs* has different effects on biofilm formation compared to a *med31*Δ mutant. (A) Gene Set Enrichment Analysis (GSEA) plot showing differential expression of genes down-regulated in a *C. albicans med31*Δ mutant in the *tlo1*Δ/*tlo2*Δ mutant (i.e. they are upregulated in the *tlo1*Δ/*tlo2*Δ mutant) (B) Biofilm formation was assessed in the indicated strains on plastic surfaces at 37°C in the presence or absence of 5% CO_2_ as indicated. Adherent biomass determined by an XTT reduction assay. The experiment was carried out in triplicate on three separate occasions (n = 9) and error bars represent the standard deviation from the mean. *P* values above figure are the result of one-way ANOVA with Tukey's post hoc test.

Uwamahoro *et al.* [15] showed that in *C. albicans* deletion of *MED31*, encoding a middle subunit of Mediator affected cytokinesis, filamentous growth and biofilm formation. Similarly, the *C. dubliniensis tlo1*Δ/*tlo2*Δ mutant described here also grew as chains of cells, typical of mutants with defects in cytokinesis (Figure S3A). However, the *C. albicans med31*Δ mutant was capable of filamentous growth in response to serum, whereas the *C. dubliniensis tlo1*Δ/*tlo2*Δ mutant is incapable of forming true hyphae in serum. GSEA analysis of our *C. dubliniensis tlo1*Δ/*tlo2*Δ mutant transcript data identified a significant enrichment for genes that are also affected in a *C. albicans med31*Δ mutant [15]. Interestingly, while the *tlo1*Δ/*tlo2*Δ and the *med31*Δ deletions affected similar genes, the *C. dubliniensis tlo1Δ/tlo2Δ* mutant showed increased expression of genes that were both Med31-activated and -repressed in *C. albicans* (Figure 7A). Inspection of these differentially expressed genes identified several genes required for biofilm formation that were downregulated in the *C. albicans med31*Δ mutant and induced in the *C. dubliniensis tlo1*Δ/*tlo2*Δ mutant, including *ALS1*, *TEC1* and *SUC1*. Uwamahoro *et al.* [15] showed that reduced *ALS1* expression in the *C. albicans med31*Δ mutant was largely responsible for the defect in biofilm formation. As the *C. dubliniensis tlo1*Δ/*tlo2*Δ mutant exhibited increased expression of *ALS1*, we examined whether this mutant was affected in biofilm formation. In contrast to the *med31*Δ mutant phenotype, deletion of *TLO1* and *TLO2* in *C. dubliniensis* enhanced biofilm growth on plastic surfaces (Figure 7B). In this case, complementation with *TLO2* reduced the amount of biofilm formation more than complementation with *TLO1* (ANOVA *P*<0.05) (Figure 7B). Thus the mediator middle domain has a different effect on biofilm formation than does the mediator tail domain.

### Tlo proteins regulate a core set of Med2 regulated genes

The genetic and biochemical data presented here and by Zhang *et al.* [9] support a hypothesis that Tlo proteins are the candidal orthologs of *S. cerevisiae* Med2. In order to support this, we decided to compare the transcript profile of the *C. dubliniensis tlo1Δ/tlo2Δ* mutant and the *S. cerevisiae med2*Δ mutant to identify if there is an evolutionarily conserved core regulon controlled by these proteins [27]. Although the experimental details of the two studies vary, a comparison of all genes regulated 1.5-fold or greater in both mutants could identify a small but significant overlap in regulated genes (Figure S8). Carbohydrate metabolism genes that were downregulated in both mutants included the carbohydrate metabolism master regulator *TYE7* and genes regulating carbohydrate catabolism and glycerol biosynthesis (*PFK1, FBA1, GPH1, GDB1*). One of the signatures of the *tlo1Δ/tlo2Δ* mutant profile was the downregulation of sulphur amino acid metabolism and in *S. cerevisiae*, we could also detect down regulation of *MET6* and *MET13* as well as several other conserved genes regulating amino acid catabolism (*GCV1, GCV2, SHM2*). Both mutants exhibited a general increase in the expression of genes encoding enzymes involved in oxidation-reduction processes (Figure S8).

### ChIP supports a role for Tlo1 in transcriptional activation and repression

**Figure 8:**
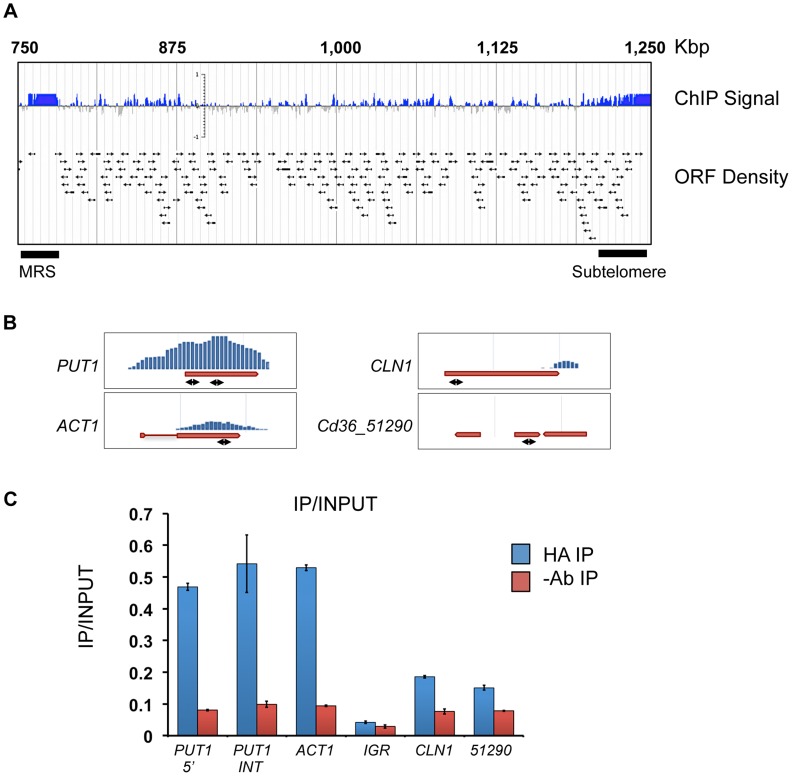
Localization of Tlo1-DNA interaction by ChIp-Chip analysis. (A) A map of the right arm of *C. dubliniensis* chromosome 5 showing Tlo1-HA enriched areas, including the subtelomeric and Major Repeat Sequence (MRS) regions. Arrows indicate the position of individual ORFs. All chromosomes can be visualised here: http://bioinf.gen.tcd.ie/jbrowse/?data=cdub. (B) ChIP-chip Enrichment profiles for *PUT1*, *ACT1*, *CLN1* and CD36_51290. Double headed arrows show approximate positions of amplimers used in QRTPCR confirmation experiments. (C) QRT-PCR confirmation of the enrichment levels shown in panel B using gene-specific primers and an intergenic region (IGR)-specific primer set (Chr5 521680–521729 bp)(Table S5). Amplification was carried out on immunoprecipitated chromatin in anti-HA antibody samples (HA-IP) and no antibody (-AB IP) negative control samples. Relative enrichment values refer to the quantity of DNA amplified from anti-HA immunoprecipitated samples (‘enriched’) relative to total input chromatin (‘non-enriched’).

In order to characterise Tlo1-DNA interactions, we carried out chromatin immunoprecipitation and high-density *C. dubliniensis* microarray (ChIP-chip) of HA-tagged Tlo1 from cells grown in YEPD broth at 30°C. On a genome wide level, we found extensive binding of Tlo1 to all chromosomes. The regions exhibiting the greatest enrichment included subtelomeric and telomeric regions and the Major Repeat Sequence (MRS) (Figure 8A). Outside of these regions, the interaction of Tlo1 was closely associated with the coding regions of genes (Figure 8A). Excluding subtelomeric regions, only 45 intergenic regions exhibited a significant enrichment peak not associated with a coding region. In addition, we did not observe any association between Tlo1 and 57 putative Pol III transcribed tRNAs and snRNAs. Tlo1 was enriched at 1,617 ORFs in the *C. dubliniensis* genome (27% of all nuclear genes; Ringo Peak Score 0.8).

Due to recent discussions in the literature regarding artefacts in ChIP data [28]–[30], we restricted detailed analysis to a group of 367 genes that exhibited highly significant Tlo1-enrichment (Ringo peak score 0.9; Table S3). The region of Tlo-enrichment was centred on the coding region with extension into 5′ and 3′ flanking non-coding regions (Figure S9A). The level of Tlo-enrichment at the 5′ region was variable and was generally <100 bp in length, however extensions up to 1000 bp in length were observed (Figure S9B). QRT-PCR analysis of DNA generated in replicate ChIP experiments confirmed the high levels of enrichment observed at *PUT1* and *ACT1* and low enrichment levels observed at non-enriched genes such as *CLN1* and Cd36_51290 and an intergenic region on chromosome 5 (bp 521680–521729) (Figure 8B, 8C). Due to the repetitive nature of the repeat sequences in the MRS, it was not possible to accurately determine the level of enrichment in this region by QRT-PCR.

**Figure 9:**
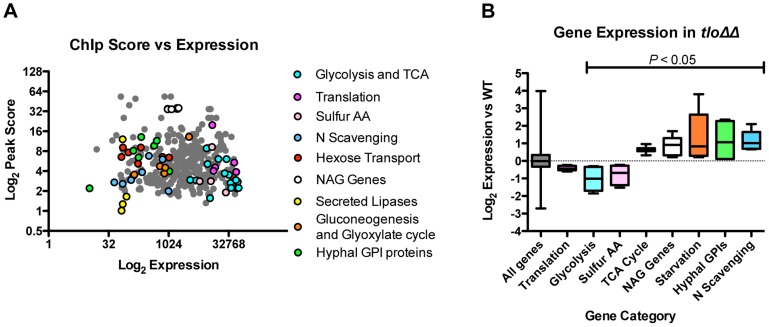
The relationship between Tlo1 binding and gene expression. (A) A plot of Tlo1 enrichment scores for the 367 most highly enriched genes versus the expression levels of those genes in YEPD broth (extracted from microarray data). The categories of highly expressed and repressed genes are color-coded; TCA = Tricarboxylic acid cycle; Sulfur AA = sulphur amino acid biosynthesis; N scavenging = Nitrogen scavenging; NAG = GlcNac metabolism; GPI = glycophosphatidylinositol anchor). (B) Analysis of the expression of Tlo1-enriched groups of genes in the wild type in the *tlo1*Δ/*tlo2*Δ (*tlo*ΔΔ) mutant indicated that the highly expressed, highly enriched genes exhibited reduced expression whereas the poorly expressed and repressed genes exhibited increased expression in the *tlo1*Δ/*tlo2*Δ (*tlo*ΔΔ) mutant. The change in expression in these groups, with the exception of the genes involved on translation, was significant (ANOVA, P<0.05).

Using fluorescence intensity data extracted from our gene expression microarrays, we compared the expression levels of the Tlo1-associated ORFs relative to non-Tlo1 associated ORFs. Tlo1-associated ORFs were found to exhibit higher expression levels (average 1.8-fold) in YEPD compared to those genes that are not occupied (Figure S9C). However, a plot of enrichment score versus expression level did not identify a direct correlation between Tlo1 enrichment and expression. Importantly, the most highly enriched genes (n = 367; Ringo peak score 0.9) covered a broad spectrum of expression levels, including genes expressed at high and low levels in YEPD (Figure 9A). We analysed the GO terms associated with these Tlo1-enriched genes and identified significant numbers of highly expressed genes associated with the GO categories glycolysis (n = 7; *P* = 0.003) and the TCA cycle (n = 7; *P* = 0.048)(Figure 9A). Manual inspection of the gene list also identified highly expressed genes involved in translation (n = 5) and sulphur amino acid metabolism (n = 4). Tlo1-enriched genes that are poorly expressed in YEPD were associated with the GO categories glyoxylate cycle (n = 3; *P* = 0.09), GlcNac catabolic process (n = 6, *P* = 0.094), amino acid catabolic process (n = 10; *P* = 0.018) and hexose transport (n = 9; *P* = 0.057). Manual inspection also identified poorly expressed genes encoding nitrogen scavenging transporters (e.g. *FRP6, MEP21, MEP22, DAL1, UGA6*) and genes involved in gluconeogenesis (*PCK1* and *FBP1*). Many YEPD repressed, hypha-specific genes were also enriched including *EED1*, *RBT1*, *HWP1*, *HWP2* and *IHD1*.

These data show that Tlo1 is present at many highly expressed metabolic genes in addition to genes normally repressed in YEPD (starvation, hyphal genes) indicating a dual role in transcriptional activation and repression. In order to support this hypothesis, we analysed the transcription of these categories of Tlo1-enriched genes in our *tlo1*Δ/*tlo2*Δ mutant. This analysis found that the highly expressed genes involved in glycolysis and sulfur amino acid metabolism exhibited significantly reduced expression (ANOVA; *P*<0.05) in the *tlo1*Δ/*tlo2*Δ mutant relative to wild type cells, indicating that Tlo1 occupancy is required for maintaining the high level of expression of these genes (Figure 9B). The exceptions to this finding were the genes encoding enzymes of the TCA cycle, which although highly expressed in wild-type cells, exhibited increased expression in the *tlo1Δ/tlo2Δ* mutant. In addition, highly Tlo1-occupied, low expression genes exhibited increased expression in the *tlo1*Δ/*tlo2*Δ mutant, including genes involved in starvation responses, nitrogen scavenging mechanisms, *N*-acetyl glucosamine catabolism and the hyphal GPI-anchored proteins (Figure 9B). These data indicate that Tlo1 and the Mediator complex are involved in both transcriptional activation and repression of different classes of genes. The fact that Tlo1-enriched genes exhibit regulation in the *tlo1*Δ/*tlo2*Δ mutant also provides support for hypothesis that Tlo proteins interact with these ORFs.

## Discussion

Despite the importance of Mediator in transcriptional regulation, few studies have investigated the role of Mediator in regulating virulence-associated characteristics in pathogenic eukaryotic microrganisms. The discovery of a widely dispersed telomeric gene family with homology to Med2 in the pathogenic yeast *C. albicans* has accelerated the level of interest in Mediator in this fungus. Published data suggest important roles for Mediator in regulating important biological processes in *C. albicans*, including filamentous growth (Med3), biofilm formation (Med31, Med20) and white-opaque switching (multiple subunits) [9], [15], [16]. Because functional analysis of the 15 *TLO* paralogs in *C. albicans* remains technically challenging, we turned to *C. dubliniensis*, which shares many characteristics with *C. albicans* but contains only two *TLO* orthologs, thereby providing an ideal genetic background in which to investigate the degree to which *TLO* gene function(s) have diverged between the two *TLO* paralogs. It has been hypothesized that in *C. albicans*, Mediator could form variant complexes that contain different Tlo/Med2 subunits that could be differentially recruited to activate or repress different subsets of genes in response to specific stimuli [9], [12]. *C. dubliniensis*, which has only two Tlo proteins that appear to associate primarily with Mediator, provides an invaluable tool to understand first, how different *TLO* genes may affect Mediator function and second how the presence of multiple variant *TLO* genes contributes to virulence.

In *C. dubliniensis*, *tlo1*Δ deletion mutants are defective in hyphal growth [14]. Here, we constructed a null *tlo1*Δ/*tlo2*Δ double mutant strain of *C. dubliniensis*. The *tlo1*Δ/*tlo2*Δ strain exhibits a wide range of phenotypic defects, indicating that they play a central role in global transcriptional control. Consistent with a role in regulating hyphal growth, deletion of both *TLO* genes reduced expression of many of the key regulators of morphogenesis including signal transducers (*RAS1, RIM101*) transcriptional regulators (*EFG1, UME6*) and kinases (*HGC1*), resulting in largely pseudohyphal growth phenotypes with reduced expression of key hyphal genes (*SOD5, RBT5, HWP1*). The *tlo1*Δ/*tlo2*Δ mutant also exhibited increased susceptibility to oxidative stress and reduced growth in the presence of alternative carbon sources. These data demonstrate that the *TLO*s of *Candida* spp. have evolved to regulate many key processes, including those important for virulence and growth in the host. In addition, the importance of Tlo in mediating transcriptional responses important for pathogenicity is shown in the similarity of our transcript profiles to those observed during infection of RHE and bone marrow-derived macrophages (Figure S6) [25], [26]. Indeed, a *tlo1*Δ/*tlo2*Δ mutant grown in YEPD mounts a transcriptional response akin to that observed following phagocytosis by macrophages [1]. Recent studies have also shown that Mediator is an important regulator of the *Candida*-macrophage interaction [31]. One key observation regarding the transcriptional changes observed in the *tlo1*Δ/*tlo2*Δ mutant was the alteration in the kinetics of induction of hypha-specific transcripts. Complementation of the *tlo1*Δ/*tlo2*Δ mutant with Tlo1 resulted in more rapid and higher levels of transcriptional induction of *SOD5*, *UME6* and *GAL10*. Some induction of these transcripts could be observed in the *tlo1*Δ/*tlo2*Δ mutant, however the amplitude of the response was greatly retarded relative to the complemented strains. These data suggest that Tlo proteins play an important role in maximizing the speed and magnitude of transcriptional changes in response to environmental cues. This characteristic is likely to be of great importance in the host where rapid responses could be important for survival, e.g. following phagocytosis or translocation to a different part of the gastrointestinal tract.

In *S. cerevisiae*, the Mediator tail regulates stress responses and the metabolism of carbohydrates and amino acids [23], [24], [27]. A direct comparison of the genes regulated in our *tlo1*Δ/*tlo2*Δ mutant and an *S. cerevisiae med2*Δ mutant supports this evolutionarily conserved role for Mediator tail in eliciting transcriptional responses to deal with changing environments [27]. In *S. cerevisiae* this effect has been shown to be centred on SAGA-regulated genes [24].

In this study, we also explored the relationship of Tlo proteins with other subunits of Mediator. A defect in filamentous growth was described by Zhang *et al.* in *C. albicans* following deletion of *Med3*, encoding another Mediator tail subunit that interacts with Tlo proteins [9]. Biochemical analysis of the Mediator complex in the *tlo1*Δ/*tlo2*Δ null mutant and the *C. dubliniensis med3*Δ mutant revealed the absence of Med5 and Med16 in Mediator purified from both mutants, suggesting that the entire tail complex is destablilized by loss of Tlo or Med3 proteins. Consistent with this idea, the *C. dubliniensis med3*Δ and *tlo1*Δ/*tlo2*Δ null mutants exhibited remarkably similar phenotypes and transcriptional profiles when cultured in YEPD. However, in response to hyphal stimulating conditions, many differences in transcriptional responses could be observed in the *tlo1*Δ/*tlo2*Δ strain and the *med3*Δ mutant strain (Figure 6), suggesting that Mediator complexes with different tail substituents may exist *in vivo* in response to specific environmental conditions. Alternatively, the deletion of either Med3 or Tlo protein may release the remaining Tail module Mediator subunits to form a sub-complex that can interact with cellular components independently of the remainder of the complex.

We next examined the relationship of Tlo with the Mediator middle subunit, Med31, described by Uwamahoro *et al.* [15]. The *C. dubliniensis tlo1*Δ/*tlo2*Δ null mutant exhibited a defect in cell separation and *CHT3* expression, which is consistent with the defect in cytokinesis described in the *C. albicans med31* mutant by Uwamahoro *et al.* [15]. These data support previous studies that suggest that regulation of cytokinesis and possibly Ace2-regulated genes is a conserved function of mediator in yeast [15], [32]. However, a *med31*Δ mutant was still capable of true hyphal growth in liquid medium whereas the *tlo1*Δ/*tlo2*Δ null mutant was completely unable to form true hyphae under the same conditions. The most striking difference in phenotype was the increased ability of the *C. dubliniensis tlo1*Δ/*tlo2*Δ mutant to form biofilm. The *C. albicans med31*Δ mutant exhibits reduced biofilm growth relative to wild-type and decreased expression of the biofilm regulator *TEC1* and the adhesin *ALS1* (Figure 7A). The *C. dubliniensis tlo1*Δ/*tlo2*Δ null mutant exhibited increased expression of many *med31*Δ downregulated genes, including *TEC1* and *ALS1*, although the roles of these genes in biofilm formation in *C. dubliniensis* have yet to be confirmed. These data illustrate the very different and possibly opposing roles that mediator tail and middle subunits may have in transcriptional regulation in *Candida *spp.

Our ChIP analysis of Tlo1 for the first time indicates the level of Tlo and Mediator occupancy across the *Candida* genome. Although Mediator tail subunits are unlikely to interact directly with DNA, they associate closely with DNA-bound transcription factors and histones and have been successfully isolated in association with DNA in other yeasts [8], [33]. We identified extensive Tlo1 binding across all *C. dubliniensis* chromosomes. Tlo1 was closely associated with Pol II transcribed ORFs as well as the telomeres and the MRS. Binding of Mediator to telomeres and sub-telomeric genes has been described in *S. cerevisiae* and Mediator has been shown to have an additional role in heterochromatin maintenance [33], [34]. Our data suggests that Mediator could play a similar role in *Candida *spp. The significance of Tlo1-enrichment at the MRS is more difficult to explain as the function of this repeat element is unknown. Further studies will be required to verify this interaction.

In order to limit the numbers of possible ChIP artefacts, often associated with highly expressed genes, we have used a very stringent cut off to define highly enriched genes. This Tlo1-enriched gene set includes repressed and moderately expressed genes, supporting the assumption that our data set is not biased for highly expressed ORFs. In addition, the highly expressed genes identified in our ChIP analysis are regulated following deletion of Tlo, supporting a direct, functional interaction with these ORFs.

The association of Mediator subunits with the coding regions of Pol II transcribed ORFs has been observed in Mediator ChIP localization experiments in other yeasts and suggests that Tlo1 may be involved in transcript elongation or in chromatin interactions at coding regions [35], [36]. We observed that although Tlo1-occupied genes on average were expressed higher than non-occupied genes (average 1.8-fold), Tlo1 was associated with ORFs having a range of expression levels. To further investigate the significance of Tlo1 ORF occupancy and Tlo1-dependent gene expression, we compared the regulation of specific classes of Tlo1 occupied genes in our *tlo1*Δ/*tlo2*Δ mutant. Highly expressed genes encoding enzymes involved in amino acid synthesis, glycolysis and translation required Tlo1 to maintain high levels of expression. In contrast, genes that exhibited low levels of expression under the conditions analysed were repressed by Tlo1. These data suggest a dual role for Tlo1 in mediating transcriptional activation and repression. Specifically, Tlo1 was required to maintain transcription of genes involved in glycolysis and translation and to repress starvation responses (glyoxylate cycle, gluconeogenesis) and several hypha-specific genes. Localisation of Mediator to both expressed and repressed genes has been observed in *S. cerevisiae* and the ability of the complex to carry out opposing regulatory functions is likely due to its ability to adopt different modular conformations [36]. Specifically, the kinase module in *S. cerevisiae*, consisting of Cdk8, Cyclin C, Srb8 and Srb9 has been shown to associate with Mediator to repress transcription [36]. A specific role for the kinase module in transcriptional repression has yet to be determined in *Candida *spp.

The *C. dubliniensis* genome harbors two *TLO* orthologs, two alleles of *TLO1* and one allele of *TLO2*. One of the central goals of our study was to determine whether different *TLO* genes could regulate different processes. To compare the effects of single copies of *TLO1* and *TLO2*, we complemented the *tlo1*Δ/*tlo2*Δ mutant independently with either *TLO1* or *TLO2*. Each of the *TLO* genes complemented *tlo1*Δ/*tlo2*Δ mutant phenotypes, albeit to different degrees; *TLO1* was better at restoring true hyphal growth and growth in galactose. *TLO2* supressed biofilm growth to a greater extent than *TLO1*. With regard to *TLO2*, it should be noted that the level of expression of the reintegrated allele was significantly higher than the wild-type allele, which may have resulted in higher levels of Tlo2 activity. However, despite this, the reintegrated *TLO2* allele did not restore filamentous growth to the same extent as wild-type *TLO1*.

Support for these functional differences was obtained when the transcript profiles of the *TLO1* and *TLO2* complemented strains were compared. It was observed that *TLO1* could restore *GAL* gene induction to a greater extent than *TLO2*. During hyphal growth, *TLO1* also restored expression of *UME6* and *SOD5* to a greater degree than *TLO2*. In contrast, during growth in YEPD *TLO2* was required for repressing transcription of aberrantly expressed hyphal genes such as *IHD1*, *SAP7* and *EED1* and the negative regulators of hyphal growth *SFL1* and *NRG1*. *TLO2* was also required for suppression of many starvation induced genes involved GlcNac metabolism (*NAG3, NAG4*) and amino acid metabolism (*CHA1, GDH3*). The data suggest that the two *TLO* paralogs in *C. dubliniensis* have evolved to regulate overlapping as well as distinct subsets of genes. Under standard growth conditions, both *TLO1* and *TLO2* are expressed in *C. dubliniensis*, suggesting that two distinct pools of Mediator exist at any one time, with the ability to regulate different subsets of genes. The implications of our study for *C. albicans* biology, where there are potentially 15 different Tlo-Mediator complex varieties with differing transcriptional activating activities, are immense. It is tempting to speculate that this variety in Mediator activity, or a varying pool of non-Mediator associated Tlo protein, could contribute to the phenotypic plasticity and adaptability of this fungus and may go towards explaining why its incidence as a commensal and opportunist is far greater compared to its close relatives *C. tropicalis* and *C. dubliniensis* which do not have this expanded repertoire of Med2 paralogs.

Over the last decade, a wealth of studies have described the global transcriptional responses of *C. albicans* to environmental stress, pH changes and nutrient availability amongst others. It is clear from these studies that one of the characteristics of *C. albicans* is its ability to rapidly respond to environmental change by mediating rapid transcriptional responses. The current study suggests a vital role for the Tlos in mediating the speed and scale of these responses, which are associated with characteristics required for commensalism (nutrient acquisition, metabolism) and pathogenicity (starvation responses, filamentation) indicating a key role for Tlo-regulated responses in the lifestyle of *C. albicans*. This, coupled with the potential diversity in the activity of the different Tlo paralogs may have contributed to the ability of *C. albicans* to colonise many diverse niches and thus to evolve to be a highly successful commensal and pathogen.

## Materials and Methods

### *Candida* strains and culture conditions

All *Candida* strains were routinely cultured on yeast extract-peptone-dextrose (YEPD) agar, at 37°C. Solid Lee's medium and Spider medium were used as described previously [37], [38]. Pal's agar medium was also used for chlamydospore induction as described previously [39]. For liquid culture, cells were grown shaking (200 rpm) in YEPD broth at 30°C or 37°C. In order to determine the doubling time of each strain, the optical densities (600_nm_) of cultures in the exponential phase of growth were plotted and analysed using the exponential growth equation function in Graphpad Prism (GraphPad, CA, USA). Doubling times and r^2^ values in Table 1 were calculated from three replicate growth curves. Glucose (2% w/v) was substituted with galactose (2% w/v) where indicated. Hyphal induction was carried out in sterile Milli-Q H_2_O supplemented with 10% (v/v) fetal calf serum at 37°C. Samples were randomized and the proportion of germ-tubes or true-hyphae in 300 cells was assessed at intervals by microscopic examination of an aliquot of culture with a Nikon Eclipse 600 microscope (Nikon U.K., Surrey, U.K.). Experiments were performed on at least three separate occasions.

Genotypes of strains used in this study are listed in Table S4. Gene disruption of *TLO1* (Cd36_72860) and *TLO2* (Cd36_35580) was achieved through use of the *SAT1* flipper cassette system [40]. Deletion constructs were created by PCR amplifying the 5′ flanking regions of *TLO1* or *TLO2* with the primer pairs CTA21KF/CTA1X and CTA22KF/CTA2X respectively and the 3′ flanking regions with the primer pairs CTA1S/CTA21SIR and CTA2S/CTA22SIR, respectively (Table S4). Ligation of these products in the corresponding restriction sites in plasmid pSFS2A resulted in the construction of plasmids pTY101 (*TLO1*) and TY102 (*TLO2*) as described [19]. The deletion construct was used to transform *C. dubliniensis* Wü284 as described previously [19] and deletion of genes was confirmed by Southern blotting (Figure S1). A single transformation with TY102 was sufficient to delete *TLO2* in strain Wü284 which was found to have a truncation in one copy of ChrR, resulting in the presence of only one copy of *TLO2*. Deletion of *MED3* was carried out using a primer tailing method with the primers MED3M13F/MED3M13R as described [41]. Reintroduction of wild-type *TLO* genes or *MED3* was achieved by PCR amplification of the entire ORF plus their upstream and downstream regulatory sequences using primer pairs CdTLO1FP/CdTLO1RP for *CdTLO1*, CdTLO2FP/CdTLO2RP for *CdTLO2* and MED3FP/MED3RP for *MED3* (Table S5) and ligation of these into the *C. dubliniensis* integrating vector pCDRI to yield plasmids pCdTLO1, pCdTLO2, and pCdMED3 respectively. These plasmids were transformed as described previously [19].

### Biochemical analysis of mediator

Mediator was purified from *C. dubliniensis* strains containing Mediator subunits tagged at their C-terminus with either HA or 6His-FLAG. *CdMED8*- and *CdTLO1*-tagging cassettes were amplified from pFA-3HA-SAT1 or pFA-6His3Flag-SAT1 using the primer pairs pZL420/pZL421 and pZL422/pZL423, respectively, as described previously [9]. Whole-cell extracts were made from strains that were untagged or had a single copy of Tlo1 or other Mediator subunit tagged with a HA or 6His-FLAG and subjected to multiple chromataographic separations as described previously [9]. The elutions from the immobilized metal affinity chromatography (IMAC; Talon Kit, Clontech Laboratories, CA, USA) step were analyzed by SDS-PAGE as indicated and proteins were revealed by staining with silver as described previously [9].

### Induction of biofilm formation

Biofilm mass was determined using an XTT reduction assay to assess metabolic activity. Prior experiments with planktonic cells indicated that wild-type and *tlo*Δ mutant strains exhibited similar rates of XTT reduction. To induce biofilm growth, a suspension of 1×10^6^ cells of each strain was prepared in 10% (v/v) foetal calf serum from 18 h cultures grown in YEPD broth at 30°C with shaking at 200 rpm A 100 µl volume of each suspension was added to triplicate wells of a 96-well, flat-bottomed polystyrene plate (Greiner BioOne) with lid. Plates were then incubated for 24 h or 48 h in a static incubator (Gallenkamp) set to 37°C. Growth in the presence of 5% (v/v) CO_2_ was carried out in a static tissue culture incubator with 5% (v/v) relative humidity (Gallenkamp). All wells were then washed 5 times with 200 µl sterile PBS to remove non-adherent cells. After the final wash, 200 µl of 200 µg/ml XTT supplemented with 50 µg/ml CoEnzyme Q (Sigma-Aldrich) was added to each well and incubated at 37°C in the dark for 50 min. A 100 µl aliquot of this suspension was transferred to a fresh plate and the absorbance measured at 480 nm using a Tecan Plate Reader system (Tecan). Results were analysed and graphed using Microsoft Excel (Microsoft).

### Transcriptional profiling with oligonucleotide microarrays

The *C. dubliniensis* whole genome oligonucleotide microarray used in this study was previously described [20]. RNA was extracted from cells grown to an OD 600 nm of 0.8 in YEPD broth at 37°C or from cells (1×10^6^) inoculated in 10% (v/v) fetal calf serum at 37°C. Cell pellets were snap frozen in liquid N_2_ and disrupted using the Mikro-Dismembrator S system (Sartorius Stedim Biotech, Göttingen, Germany). RNA was prepared using the Qiagen RNeasy mini-kit. A 200 ng aliquot of total RNA was labelled with Cy5 or Cy3 using the Two-Color Low Input Quick Amp labeling Kit (Agilent Technologies) according to the manufacturer's instructions. Array hybridization, washing, scanning and data extraction was carried out in GenePix Pro 6.1 (Axon) as described [20]. For each condition, four biological replicate experiments were performed, including two dye swap experiments. Raw data were exported to GeneSpring GX12 and signals for each replicate spot were background corrected and normalized using Lowess transformation. Log_2_ fluorescence ratios were generated for each replicate spot and averaged. Genes differentially expressed (1.5-fold) relative to wild-type were identified from those that passed a *t*-test (*P*≤0.05) and satisfied a post-hoc test (Storey with Bootstrapping) with a corrected *Q* value ≤0.05. Hierarchical clustering was used to compare gene expression in each condition using the default settings in Genespring GX12. The Gene Set Enrichment Analysis (GSEA) PreRanked tool (available to download at www.broadinstitute.org/gsea/index.jsp) was used to investigate whether our data sets were enriched for particular genes present in published data sets [42]. This analysis required the use of a database of publicly available genome-wide data sets, constructed by Andre Nantel (National Research Council of Canada, Montreal), that can be downloaded from the Candida Genome Database (CGD). This database included 171 lists of up- and down-regulated genes from microarray experiments, *in vivo* promoter targets derived from ChIP-chip experiments performed on 36 transcription factors, members of 3,601 Gene Ontology (GO) term categories, and 152 pathways, as curated by CGD, amongst others, and is fully described by Sellam *et al.* [43]. Genes in our expression data sets were first ‘ranked’ based on Log2 values from highest to lowest. The GSEA PreRanked tool was then used to determine if particular gene sets in the database were enriched towards the top or bottom of this ranked list. Enrichment plots show the level of enrichment towards the top (red) or bottom (blue) of the ranked list. The normalized enrichment score (NES) indicates the level of enrichment at the top (positive NES) or bottom (negative NES) of the ranked list and is supported by a p value and a False Discovery Rate (FDR) value to indicate the likelihood of false positive results.

Microarray data have been submitted to Gene Expression Omnibus (GEO), accession number: GSE59113.

### Real-time PCR analysis of gene expression

RNA for QRT-PCR was isolated using the RNeasy Mini-kit (Qiagen). Cells were disrupted using a FastPrep bead beater (Bio101). RNA samples were rendered DNA free by incubation with Turbo-DNA free reagent (Ambion, Austin, TX). cDNA synthesis was carried out using the Superscript III First strand synthesis sytem for RT-PCR (Life technologies) as described by Moran et al. [19] Reactions were carried on an ABI7500 Sequence Detector (Applied Biosystems, Foster City, CA) using MicroAmp Fast Optical 96-well Reaction plates (Applied Biosystems) in 20 µl reactions using 1X Fast SYBR Green PCR Master Mix (Applied Biosystems), 150 nM of each oligonucleotide (Table S4) and 2 µl of diluted template (10 ng). Cycling conditions used were 95°C for 20 sec, followed by 40 cycles of 95°C for 3 sec and 60°C for 30 sec, the latter of which was the point of detection of fluorescence. This was followed by a melt curve stage as a quality control point. Gene expression levels were normalized against the expression levels of the constitutively expressed *ACT1* gene in the same cDNA sample. Gene specific primers are shown in Table S5. Each gene-specific set was shown to amplify at a similar efficiency (within 10%) to the control *ACT1* primer set in primer optimization experiments.

### Chromatin immunoprecipitation and microarray hybridisation (ChIP-chip)

*C. dubliniensis* strain Tlo1-HA, derived from strain Wü284, was grown at 30°C degrees and harvested at an OD 600 nm of 2.0. A secondary cross-linking strategy was employed to fix chromatin that involved initial treatment of cells with dimethyl adipimidate (10 mM) for 45 min with agitation at room temperature followed by cross-linking with 1% formaldehyde. Cells were washed twice in PBS prior to spheroplasting in 5 ml of spheroplasting buffer (1.2 M sorbitol, 20 mM 4-(2-hydroxyethyl)-1-piperazineethanesulfonic acid [HEPES] pH 7.4) containing 0.4 mg/ml Zymolyase 100T (Seikagaku Corp., Japan) and incubated for 2.5 h 30°C with rotation. DNA isolation and fragmentation was carried out as described in Ketel *et al* [44] using a Branson sonifier to yield DNA fragments of 500–600 bp. Chromatin immunoprecipitation (ChIP) was carried out as described by Ketel et al. [44] using an anti-HA mouse monoclonal antibody (clone 12CA5) which was incubated overnight at 4°C on a rotating wheel. The following day, 70 µl of Protein A agarose beads (Santa Cruz Biotech, California, USA) were added and mixed at 4°C for 4 h. DNA was recovered as described by Ketel et al [44] and resuspended in 80 µl TE buffer for downstream applications.

Microarray chip analysis (CGH Microarray) was carried out as described in the Agilent Yeast ChIP-on-chip analysis protocol handbook (Version 9.2, 2007). Competitive hybridization was carried out on a whole genome *C. dubliniensis* oligonucleotide microarray consisting of 175,758 unique 60mer probes, spaced at 20 bp intervals (Agilent Technologies). Experiments were performed with triplicate biological replicate samples. The IP samples were labelled with Cy3 and input samples (total lysate control) were labelled with Cy5. One sample was dye swapped. Array hybridisation and washing were carried out using standard Agilent Technologies CGH hybridisation buffers and washes. Scanning of the array was carried out on an Agilent G2565B rotating multi-slide hyb-scanner (Agilent Technologies) using a 5 µm resolution and PMT settings at 100% for both channels (Green/Red). The scan area was set to 61×21.6 mm. The raw images were processed using Agilent Feature Extraction software suite v 10.5.1.1. and feature extraction was automatically carried out using the ‘CGH automatic’ programme to align and normalise fluorescent probe spots on the array.

Data were read into the Bioconductor [45] package Ringo [46] and pre-processed with the “nimblegen” method. This was followed by merging of replicates and smoothing with a window half size of 500 bp. ChIP-enriched regions were calculated with an enrichment threshold based firstly on a 0.8 percentile, followed by a more stringent 0.9 percentile which was used for the analysis presented here. Genes were assigned to an enrichment region if they overlapped up to 500 bp away from the gene boundaries. For visualisation of the data a JBrowse [47] instance was set up on http://bioinf.gen.tcd.ie/jbrowse/?data=cdub. The ChIP data have been submitted to GEO, accession number GSE60173.

## Supporting Information

Figure S1 Southern immunoblots of *Nde*I digested genomic DNA from *C. dubliniensis*. Hybridizing fragments were detected using a digoxigenin-labeled probe homologous to bases +51–250 of *TLO1* (100% homology) and bases +51–248 of *TLO2* (87% homology). *TLO1* and *TLO2* hybridizing Fragments were predicted to be 14.07 Kb and 5.96 Kb in size. Only one copy of *TLO2* was detected in Wü284, hence the absence of a second hybridizing allele in lane 3 (*tlo2*Δ).
(TIFF)

Figure S2 Immunoblotting of whole cell extracts of *C. dubliniensis* using an anti-HA antibody for detection Tlo1-HA, Med8-HA and Med3-HA. Whole-cell extracts were made from strains that were untagged or had a single copy of a Tlo or other Mediator subunit tagged with the HA epitope. The strain used for each extract is listed above the lanes. Multiple volumes were loaded for each strain to allow normalization with an anti-tubulin antibody.
(TIFF)

Figure S3 (**A**) Microscopic appearance of *tlo1*Δ/*tlo2*Δ (*tlo*ΔΔ) mutant cells and reintegrant strains (+*TLO1* or +*TLO2*) stained with calcofluor white. (B) Colony morphology of *tlo1*Δ/*tlo2*Δ (*tlo*ΔΔ) mutants and reintegrant strains (+*TLO1* or +*TLO2*) on Spider agar medium.
(TIFF)

Figure S4 Additional phenotypes in the *tlo1*Δ/*tlo2*Δ mutant. (A) Production of pseudohyphae and chlamydospores (indicated by arrow) following growth on solid Pal's medium (B) Comparative growth on solid Lee's medium. Lee's medium was supplemented with 1% (w/v) peptone in the lower panel, as indicated.
(TIFF)

Figure S5 Phenotypes in the *C. dubliniensis med3*Δ mutant. (A) Growth of the *med3*Δ mutant in YEP-Gal. (B) Susceptibility of the *med3*Δ mutant to hydrogen peroxide.
(TIFF)

Figure S6 Enrichment plots from Gene Set Enrichment Analysis (GSEA) of the transcript profile of the *tlo1*Δ/*tlo2*Δ mutant showing enrichment for genes regulated during infection of reconstituted human epithelium (RHE, [25]) and bone marrow derived macrophages (BMDM, [26]).
(TIFF)

Figure S7 Heat map showing the expression of glycolytic and gluconeogenic (highlighted) pathway encoding genes in the *tlo1*Δ*/tlo2*Δ mutant and the *med3*Δ mutant during growth in YEPD broth and 10% serum. Each gene is represented by data from duplicate microarray spots.
(TIFF)

Figure S8 Tlo proteins regulate similar processes to Med2 in *S. cerevisiae*. Venn diagrams illustrating the numbers of orthologous genes commonly regulated in an *S. cerevisiae Scmed2* mutant and the *C. dubliniensis tlo1*Δ/*tlo2*Δ (*tlo*ΔΔ) mutants following growth in YEPD broth. *P* values were generated by hypergeometric probability testing (http://www.geneprof.org/GeneProf/tools/hypergeometric.jsp).
(TIFF)

Figure S9 (A) Composite, smoothed Tlo1-enrichment plot generated with a sliding window of 100 bp from the plots of the 367 highly-enriched genes (Ringo peak score 0.9) (B) Box-plot graph showing the distribution and length of Tlo1 enrichment at the 5′ and 3′ ends of 367 highly-enriched genes (Ringo peak score 0.9) (C) Graph showing average expression levels of Tlo1 occupied genes (n = 1613, mean 4783) and non-occupied genes (n = 3744, mean 2620) in wild-type *C. dubliniensis*. Expression data are raw, background corrected fluorescence intensity signals extracted from microarray data sets. *P* value generated with unpaired, two-tailed t-test.
(TIFF)

Table S1 List of genes exhibiting *TLO1*- or *TLO2*-specific regulation following complementation of the *tlo1*Δ/*tlo2*Δ (*tlo*ΔΔ) mutant with either *TLO1* (+*TLO1*) or *TLO2* (+*TLO2*). Transcript profiles were generated on cells grown to OD 600_nm_ 0.8 in YEPD at 37°C. Expression values are to Log_2_ ratios.
(XLSX)

Table S2 List of genes exhibiting *TLO1*- or *TLO2*-specific regulation following complementation of the *tlo1*Δ/*tlo2*Δ (*tlo*ΔΔ) mutant with either *TLO1* (+TLO1) or *TLO2* (+TLO2). Transcript profiles were generated on cells grown for 1 h in 10% serum at 37°C. Expression values are to Log_2_ ratios.
(XLSX)

Table S3 List of genes considered highly enriched for Tlo1-HA (Ringo peak score 0.9) in ChIP-chip experiments in YEPD broth. Column ‘*tlo*ΔΔ vs WT YEPD’ indicated Log_2_ gene expression ratios in the *tlo1*Δ/*tlo2*Δ (*tlo*ΔΔ) mutant relative to wild-type generated in gene expression microarrays. ‘YEPD Expression level’ refers to the raw, background corrected, Loess normalised fluorescent intensity level for that gene in gene expression microarray experiments.
(XLSX)

Table S4 Strains of *Candida *spp. used in this study.
(DOCX)

Table S5 Oligonucleotide primers used in this study.
(DOCX)
