# Supplementary figures and images for: Telomeric ORFs (TLOs) in Candida spp. Encode Mediator Subunits That Regulate Distinct Virulence Traits
Source: PLoS Genet. 2014 Oct 30;10(10):e1004658. doi: 10.1371/journal.pgen.1004658 (PMC4214616; doi:10.1371/journal.pgen.1004658)

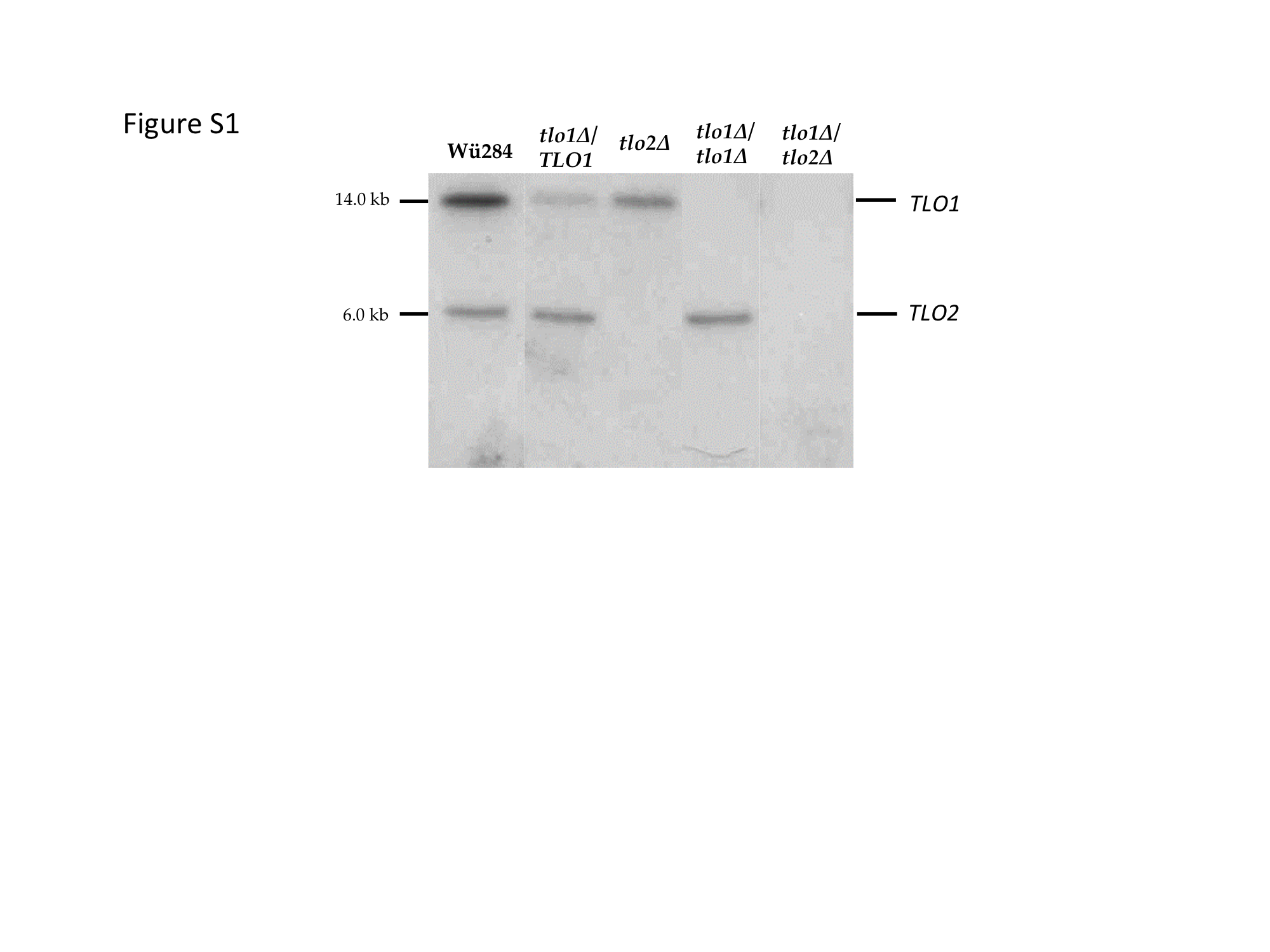

Supplement: Figure S1 — Southern immunoblots of NdeI digested genomic DNA from C. dubliniensis. Hybridizing fragments were detected using a digoxigenin-labeled probe homologous to bases +51–250 of TLO1 (100% homology) and bases +51–248 of TLO2 (87% homology). TLO1 and TLO2 hybridizing Fragments were predicted to be 14.07 Kb and 5.96 Kb in size. Only one copy of TLO2 was detected in Wü284, hence the absence of a second hybridizing allele in lane 3 (tlo2Δ). (TIFF) [file pgen.1004658.s001.tiff]

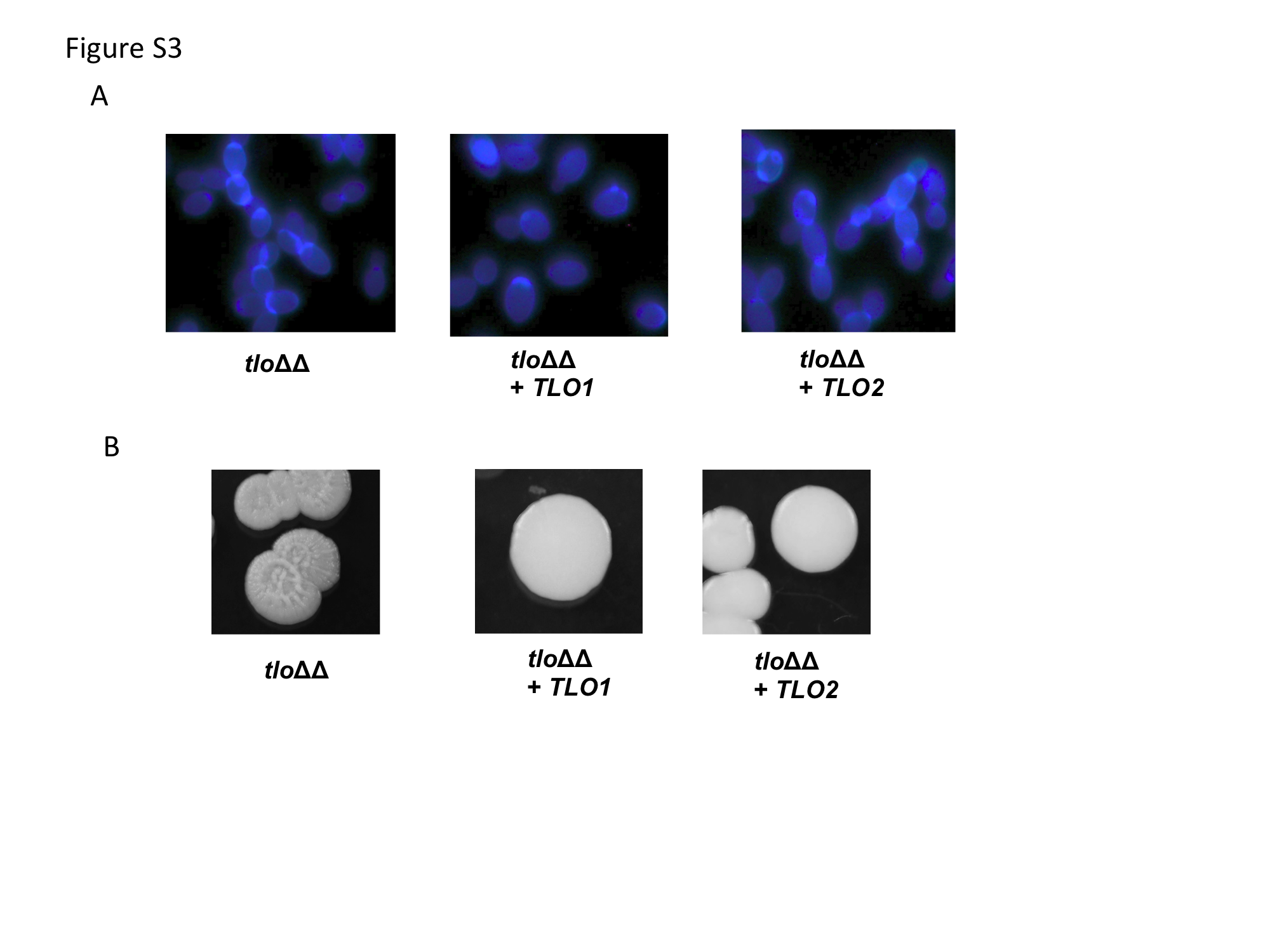

Supplement: Figure S3 — (A) Microscopic appearance of tlo1Δ/tlo2Δ (tloΔΔ) mutant cells and reintegrant strains (+TLO1 or +TLO2) stained with calcofluor white. (B) Colony morphology of tlo1Δ/tlo2Δ (tloΔΔ) mutants and reintegrant strains (+TLO1 or +TLO2) on Spider agar medium. (TIFF) [file pgen.1004658.s003.tiff]

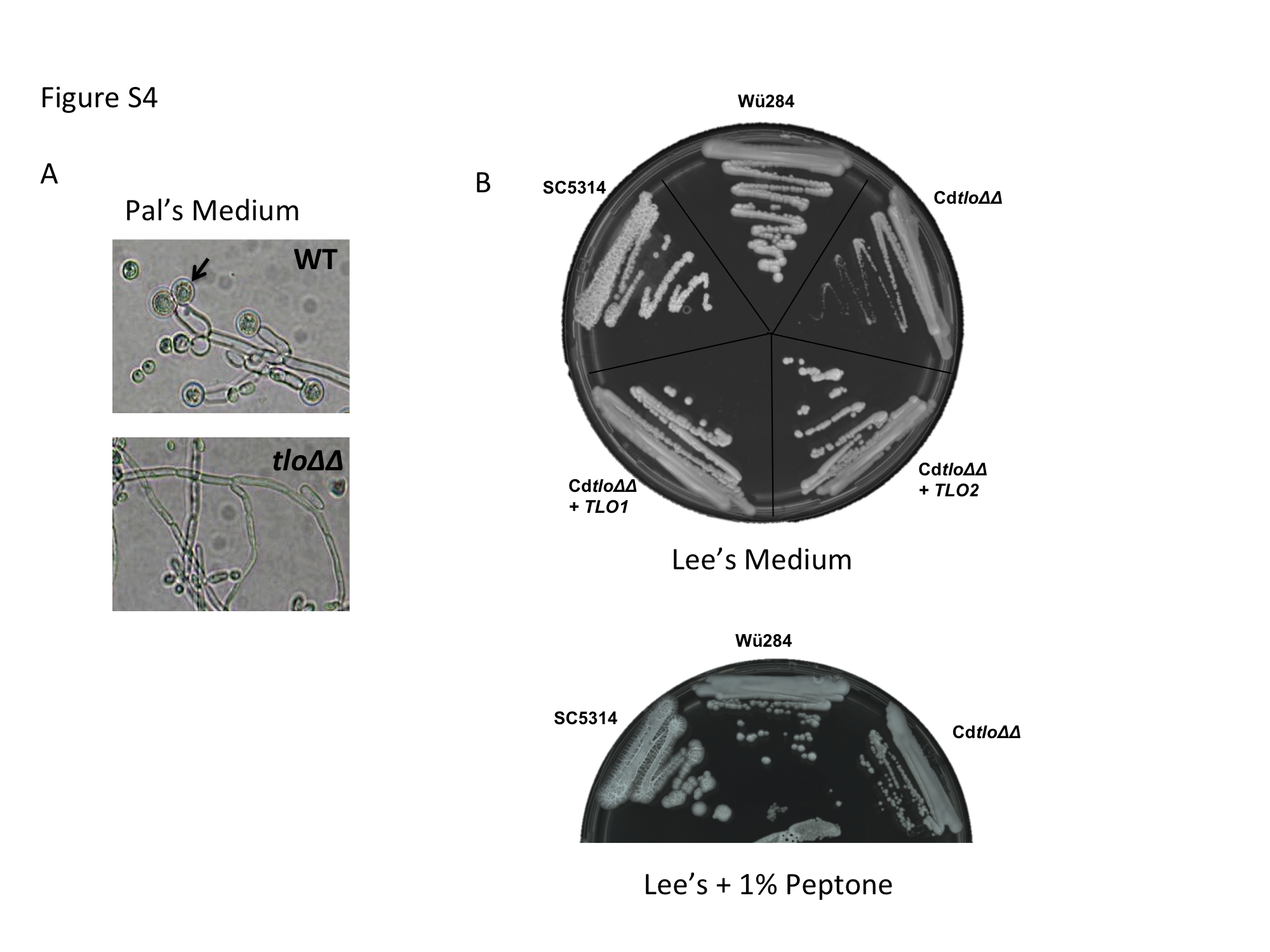

Supplement: Figure S4 — Additional phenotypes in the tlo1Δ/tlo2Δ mutant. (A) Production of pseudohyphae and chlamydospores (indicated by arrow) following growth on solid Pal's medium (B) Comparative growth on solid Lee's medium. Lee's medium was supplemented with 1% (w/v) peptone in the lower panel, as indicated. (TIFF) [file pgen.1004658.s004.tiff]

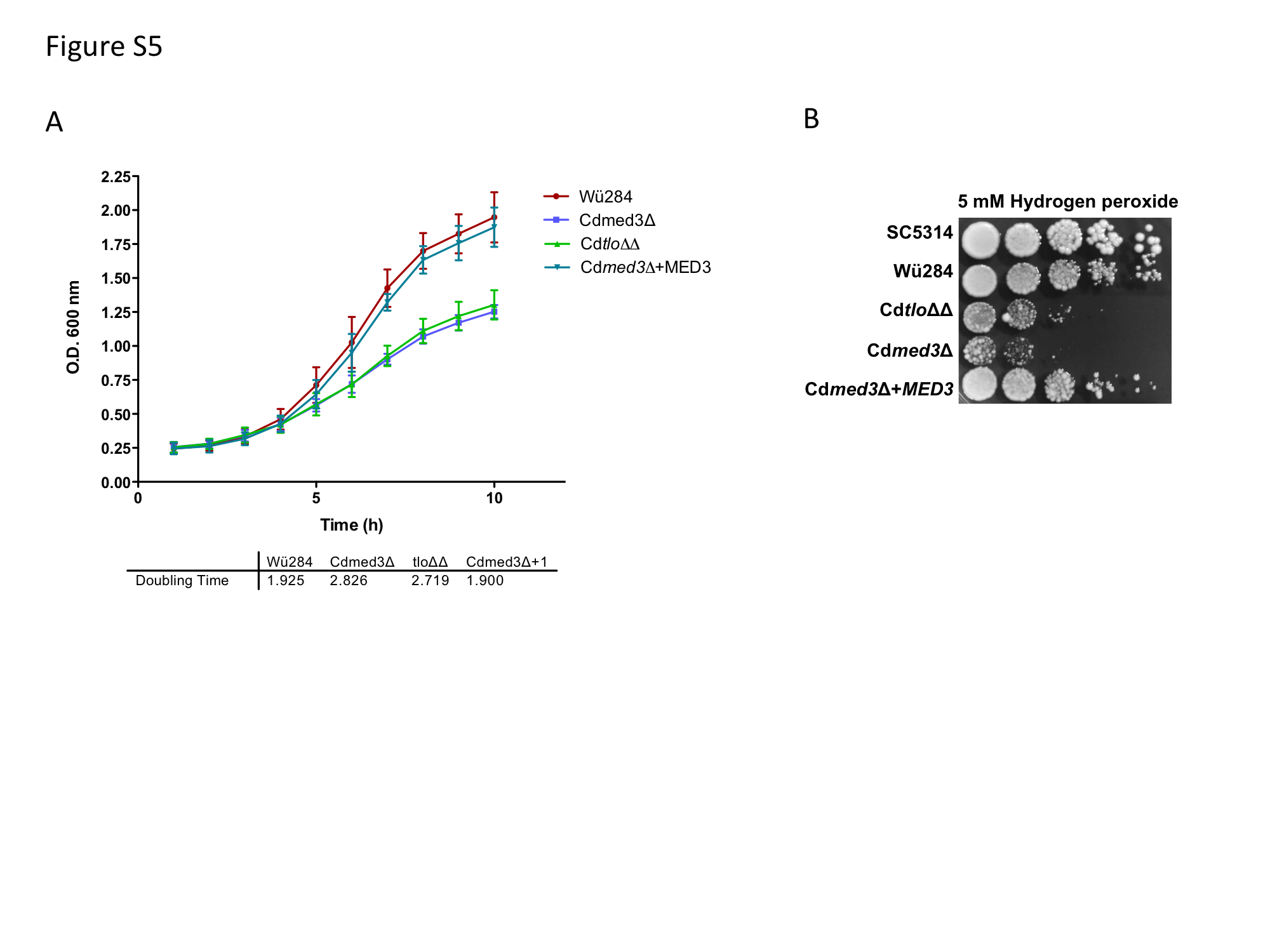

Supplement: Figure S5 — Phenotypes in the C. dubliniensis med3Δ mutant. (A) Growth of the med3Δ mutant in YEP-Gal. (B) Susceptibility of the med3Δ mutant to hydrogen peroxide. (TIFF) [file pgen.1004658.s005.tiff]

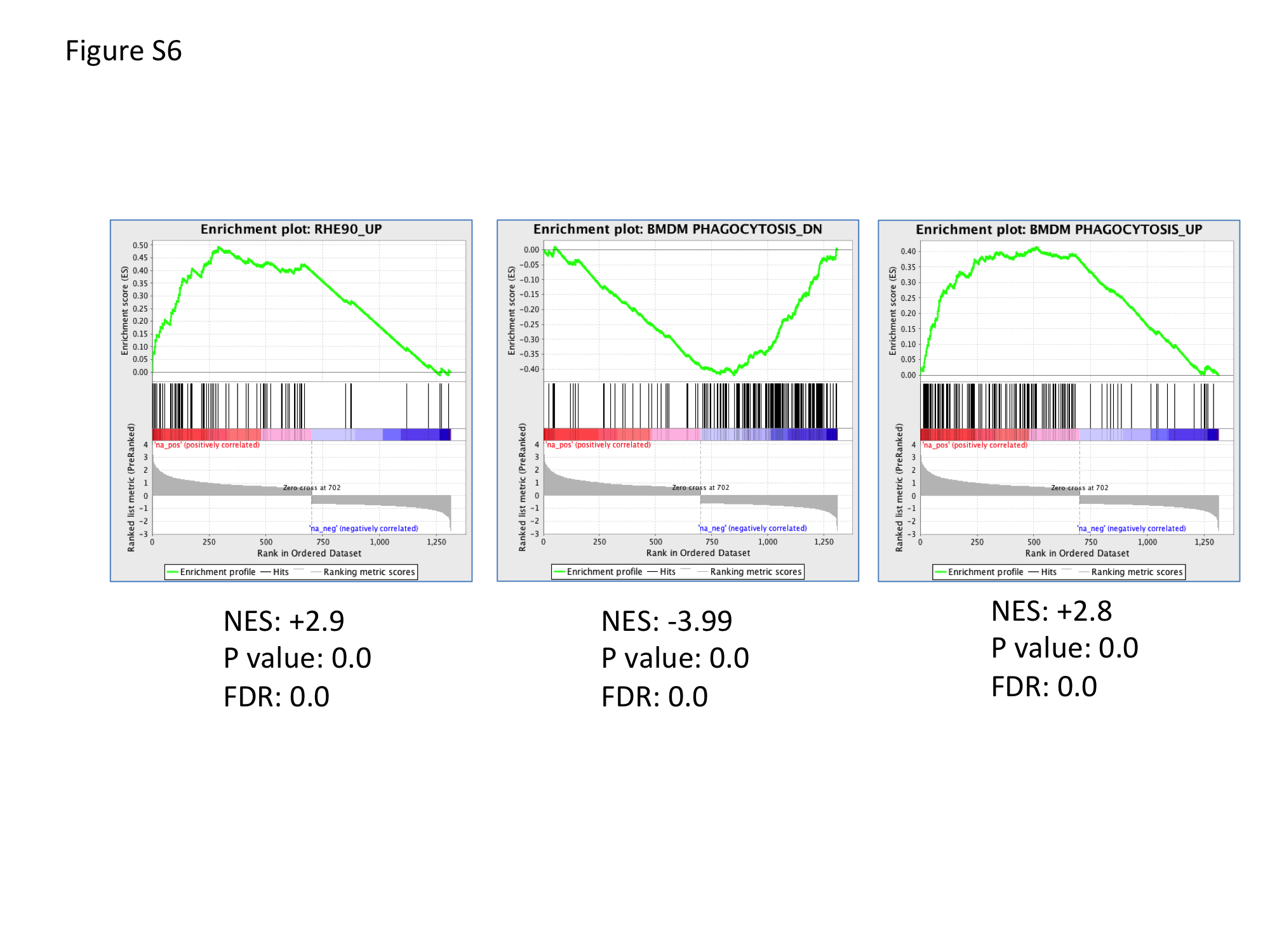

Supplement: Figure S6 — Enrichment plots from Gene Set Enrichment Analysis (GSEA) of the transcript profile of the tlo1Δ/tlo2Δ mutant showing enrichment for genes regulated during infection of reconstituted human epithelium (RHE, [25]) and bone marrow derived macrophages (BMDM, [26]). (TIFF) [file pgen.1004658.s006.tiff]

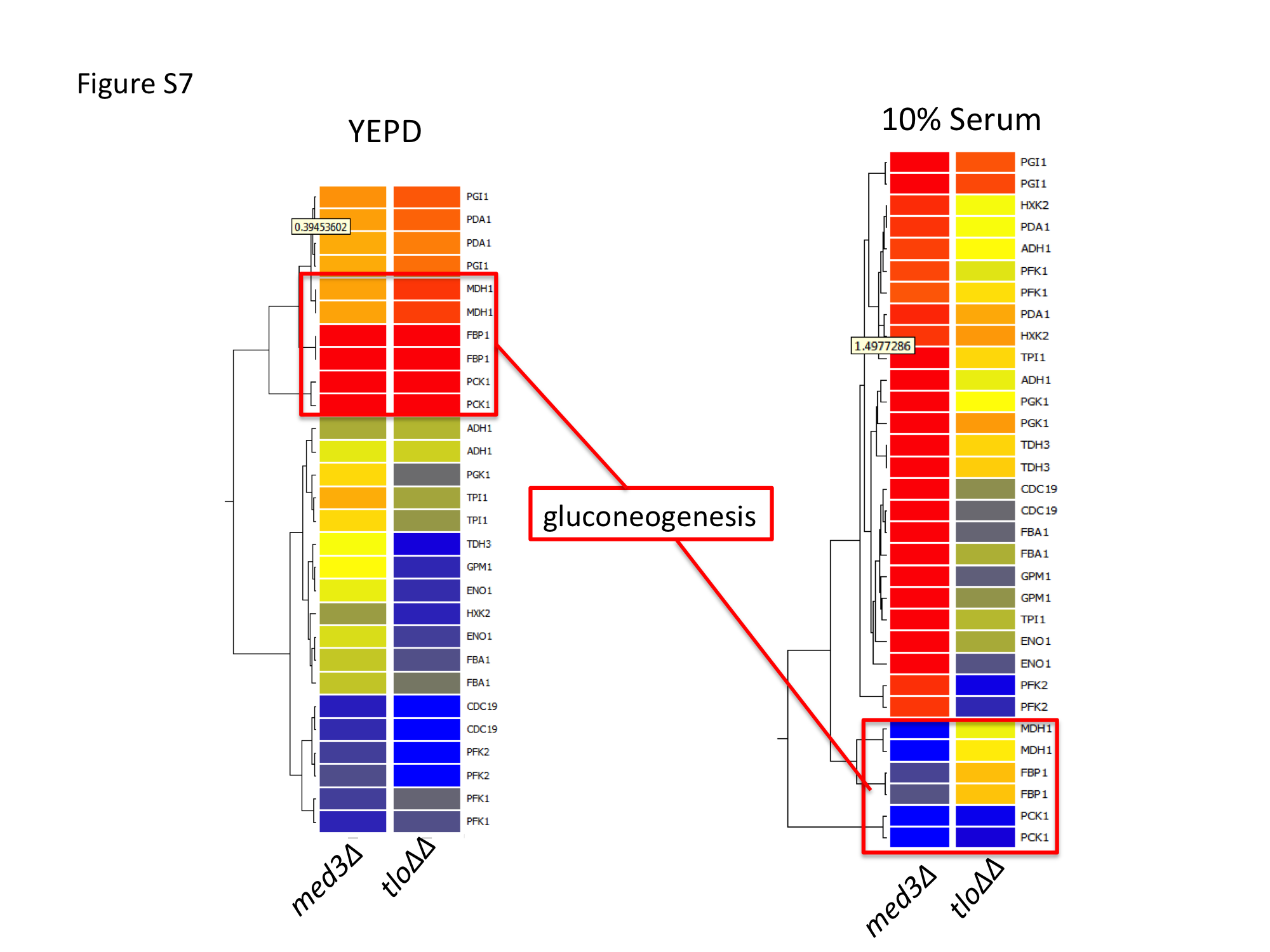

Supplement: Figure S7 — Heat map showing the expression of glycolytic and gluconeogenic (highlighted) pathway encoding genes in the tlo1Δ/tlo2Δ mutant and the med3Δ mutant during growth in YEPD broth and 10% serum. Each gene is represented by data from duplicate microarray spots. (TIFF) [file pgen.1004658.s007.tiff]

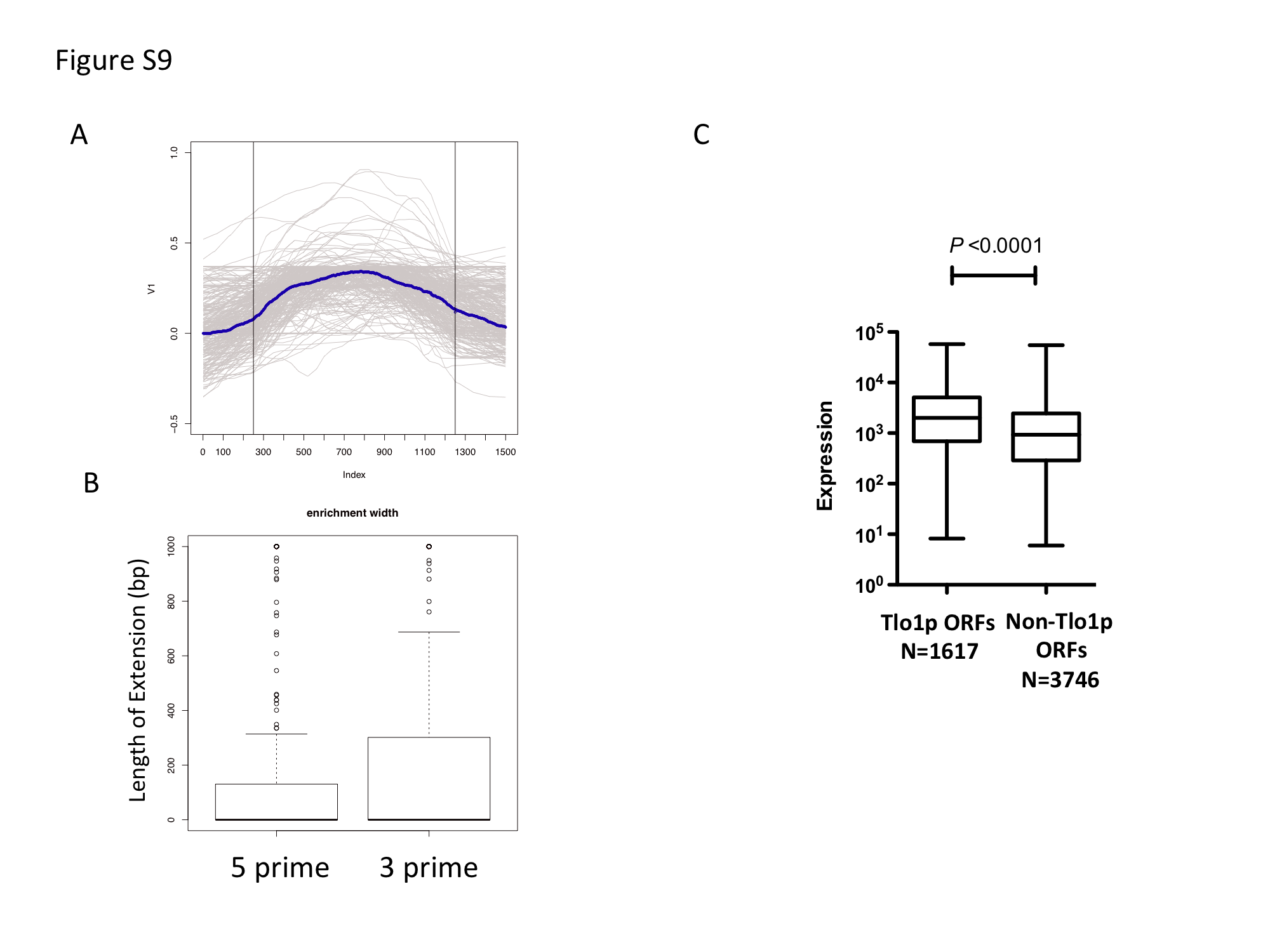

Supplement: Figure S9 — (A) Composite, smoothed Tlo1-enrichment plot generated with a sliding window of 100 bp from the plots of the 367 highly-enriched genes (Ringo peak score 0.9) (B) Box-plot graph showing the distribution and length of Tlo1 enrichment at the 5′ and 3′ ends of 367 highly-enriched genes (Ringo peak score 0.9) (C) Graph showing average expression levels of Tlo1 occupied genes (n = 1613, mean 4783) and non-occupied genes (n = 3744, mean 2620) in wild-type C. dubliniensis. Expression data are raw, background corrected fluorescence intensity signals extracted from microarray data sets. P value generated with unpaired, two-tailed t-test. (TIFF) [file pgen.1004658.s009.tiff]
