# Supplementary material for: Telomeric ORFs (TLOs) in Candida spp. Encode Mediator Subunits That Regulate Distinct Virulence Traits
Source: PLoS Genet. 2014 Oct 30;10(10):e1004658. doi: 10.1371/journal.pgen.1004658 (PMC4214616; doi:10.1371/journal.pgen.1004658)
Supplement: Table S4 — Strains of Candida spp. used in this study. (DOCX) [file pgen.1004658.s013.docx]

Table S4. Strains of *Candida* spp. used in this study.

| Strain | Genotype | Parent | Reference |
| --- | --- | --- | --- |
| SC5314 | *Candida albicans* Wild-type | n/a | 1 |
| Wü284 | *Candida dubliniensis* Wild-type | n/a | 2 |
| *tlo1*Δ/*TLO1* | *tlo1*Δ::FRT/*TLO1* | Wü284 | 3 |
| *tlo1*Δ*/tlo1*Δ | *tlo1*Δ::FRT/*tlo1*Δ::FRT | *tlo1*Δ/*TLO1* | 3 |
| *tlo2*Δ | *tlo2*Δ::FRT* | Wü284 | This study |
| *tlo*ΔΔ | *tlo1*Δ::FRT/ *tlo1*Δ::FRT; *tlo2Δ*::FRT* | *tlo1*Δ*/tlo1*Δ | This study |
| *tlo*ΔΔ*-TLO1* | *tlo1*Δ::FRT/ *tlo1*Δ::FRT; *tlo2*Δ::FRT*; *CDR1*/*cdr1*::pCDRI-*TLO1* | *tlo*ΔΔ | This study |
| *tlo*ΔΔ*-TLO2* | *tlo1*Δ::FRT/ *tlo1*Δ::FRT; *tlo2Δ*::FRT*; *CDR1*/*cdr1*::pCDRI-*TLO2* | *tlo*ΔΔ | This study |
| *med3Δ*/*MED3* | *med3*Δ::FRT/*MED3* | Wü284 | This study |
| *med3Δ* | *med3*Δ::FRT/ *med3*Δ::FRT | *med3Δ*/*MED3* | This study |
| *med3Δ*-*MED3* | *med3*Δ::FRT/ *med3*Δ::FRT; *CDR1*/*cdr1*::pCDRI-*MED3* | *med3Δ* | This study |
| Tlo1-HA | *TLO1::TLO1-HA-NatR/TLO1* | Wü284 | This study |
| Tlo1-HF | *TLO1::TLO1-6HisFLAG-NatR/TLO1* | Wü284 | This study |
| Med3-HA | *MED3::MED3-HA-NatR/MED3* | Wü284 | This study |
| Med8-HA | *MED8::MED8-HA-NatR/MED8* | Wü284 | This study |
| Med8-HF | *MED8::MED8-6His-FLAG-NatR/MED8* | Wü284 | This study |
| *tloΔ-*Med8*-*HF | *tlo1*Δ::FRT/ *tlo1*Δ::FRT, tlo2Δ::FRT/ tlo2Δ::FRT, *MED8::MED8-6His-FLAG-NatR/MED8* | *tlo*ΔΔ | This study |
| *med3Δ-*Med8*-*HF | *med3*Δ::FRT/ *med3*Δ::FRT, *MED8::MED8-6His-FLAG-NatR/MED8* | *med3Δ* | This study |

* Only one allele of *TLO2* was detected in Wü284 and derivative strains

1. Gillum, A. M., Tsay, E. Y. & Kirsch, D. R. Isolation of the Candida albicans gene for orotidine-5'-phosphate decarboxylase by complementation of S. cerevisiae ura3 and E. coli pyrF mutations. *Mol. Gen. Genet.* **198,** 179–182 (1984).

2. Morschhäuser, J., Ruhnke, M., Michel, S. & Hacker, J. Identification of CARE-2-negative Candida albicans isolates as Candida dubliniensis. *Mycoses* **42,** 29–32 (1999).

3. Jackson, A. P. *et al.* Comparative genomics of the fungal pathogens Candida dubliniensis and Candida albicans. *Genome Res* **19,** 2231–2244 (2009).
