# Supplementary material for: Telomeric ORFs (TLOs) in Candida spp. Encode Mediator Subunits That Regulate Distinct Virulence Traits
Source: PLoS Genet. 2014 Oct 30;10(10):e1004658. doi: 10.1371/journal.pgen.1004658 (PMC4214616; doi:10.1371/journal.pgen.1004658)
Supplement: Table S5 — Oligonucleotide primers used in this study. (DOCX) [file pgen.1004658.s014.docx]

**TABLE S5 Oligonucleotide primers used in this study**

| **Primer Name** | **Primer Sequence** | **Restriction Site** |
| --- | --- | --- |
| CTA21KF *(TLO1)* | ATGGGGTACCTTTCTGACAACCGCTTCACG | *Kpn*I |
| CTA1XR *(TLO1)* | ATCCCTCGAGTTGTTTGTAAGTTAGCTGAC | *Xho*I |
| CTA1SIIF *(TLO1)* | ATCGCCGCGGGAAGAGTTTGATGTAGATAG | *Sac*II |
| CTA21SIR *(TLO1)* | GGCCGAGCTCAAGAGGGACCCGACCAAGAC | *Sac*I |
| CTA22KF *(TLO2)* | ATGGGGTACCGACCCTGAAGTTGTTACGTG | *Kpn*I |
| CTA2XR *(TLO2)* | ATCCCTCGAGTCGTCGAGGGCGTCCTCTAG | *Xho*I |
| CTA2SIIF *(TLO2)* | ATCGCCGCGGACTCAAACCATTGACGACAC | *Sac*II |
| CTA22SIR *(TLO2)* | GGCCGAGCTCGACTTCACGGAAAACTTGTC | *Sac*I |
| MED3M13F | AAAGGTCAGGGAGTCCAATCGCCTACAAAAAACAAATATAAAGGGTAACCAAATGTGATACTGAGCAACCATAGTTAGATGTAAAACGACGGCCAGT | *NA* |
| MED3M13R | AAACTGTGTTATATATAATACATACATCTATATATGCTTCGAAATAAATGTTAGTGATACTACTCTGAAGAACGAGCTACTGTTACACGGAAACAGCTATGACCATG | *NA* |
| CdTLO1FP | GGATCCCTAGGCCAATTTTCATCA | *Bam*HI |
| CdTLO1RP | ACTACTAAAGCCCACAGATGACCAC | *Spe*I |
| CdTLO2FP | GGATCCTTTGGTGAGGTAAGGCCAAG | *Bam*HI |
| CdTLO2RP | ACTACTGAAATTTGTGGTGCGGAACT | *Spe*I |
| MED3FP | ATAAGAATGCGGCCGCGTAAAGCAAGTCATAATATACAA | *Not*I |
| MED3RP | GGACCGCGGCTTGTGAAGTGAGATATTTCAT | *Sac*II |
| TLO1F | CGGAAAGACAAGCGATAAGGT | *NA* |
| TLO1R | CCATCCAAGCCTCCATCAAG | *NA* |
| TLO2F | TGGTGTGTTGCCGTTATGAT | *NA* |
| TLO2R | TTTTTGTCTTTCTCCGTCTTC | *NA* |
| UME6F | TACCACCACCACCACCAT | *NA* |
| UME6R | TATCCCATTTCCAAGTCCA | *NA* |
| SOD5F | AGGGGTTGAAATGGTTCTCG | *NA* |
| SOD5R | GTCCATTCCCATATCATGTCC | *NA* |
| GAL10F | GTGCAGGCTATTGAAACTGTG | *NA* |
| GAL10R | TCACCAGGGCCAATATCTC | *NA* |
| HWP1F | CAGCTGGTATTAAAACTAACCCAGC | *NA* |
| HWP1R | ATTCTAATGTGGTTGGAATAGCACC | *NA* |
| PUT1_5F | GGGTCACCAAACCAACCAAC | *NA* |
| PUT1_5R | TCGTGCAATCTCAATCCCGT | *NA* |
| PUT1_INTF | TGAGATTGCACGAACGTGGT | *NA* |
| PUT1_INTR | GGCGGCATCTTTAGCAAACC | *NA* |
| IGF | AATTAATGAGAATTGCAGTGAGTGT | *NA* |
| IGR | AATGCATGAATGACGGATTGAA | *NA* |
| CLN1F | AACGTCGACCATATCATCCAA | *NA* |
| CLN1R | TGGTTGTAAATCAATCATGGCCG | *NA* |
| ACT1F | AGCTCCAGAAGCTTTGTTCAGACCAG | *NA* |
| ACT1R | TGCATACGTTCAGCAATACCTGGG | *NA* |
| Cd36_51290F | ACAACTGATTCCCAGTGTTCCA | *NA* |
| Cd36_51290R | AATGGAGCACGTTTCTGGGC | *NA* |
| pZL420 | TTGAAAATAACTGCTGGAGGTCAAAAAGGGTTACATCCAAATCAAGTATTGAAATTCATGTGTCAAGGTCAGATAggtcgacggatcccc | *NA* |
| pZL421 | TGTCACCTCTTTAGAATAATACAAATCACCACCTCCACCACCACCACCACAACCACCATCTTCCTACTTTCTtcgatgaattcgagctcg | *NA* |
| pZL422 | GGGGACAATAGCAACAACGACAATCAAGTCAATGAAGAGTTTGATGTAGATAGCTTCTTGAACCAATTTGGTAATggtcgacggatcccc | *NA* |
| pZL423 | ATATAATGTTGCTTATACTTGAAATACAAATGGTTGATGGTAAAGAAACGCTTTTACCCACTACCCTACACAtcgatgaattcgagctcg | *NA* |
